# Multifunctional nanoparticles confers both multiple inflammatory mediators scavenging and macrophage polarization for sepsis therapy

**DOI:** 10.1016/j.mtbio.2024.101421

**Published:** 2024-12-20

**Authors:** Wenjie Xi, Weijie Wu, Lili Zhou, Qi Zhang, Shushu Yang, Lihong Huang, Yijun Lu, Jing Wang, Xinjin Chi, Yang Kang

**Affiliations:** a Surgical Anesthesia Center, The Seventh Affiliated Hospital of Sun Yat-sen University, Shenzhen, 518107, China; b Scientific Research Center, The Seventh Affiliated Hospital of Sun Yat-sen University, Shenzhen, 518107, China; c Department of Orthopaedics, The Eighth Affiliated Hospital of Sun Yat-sen University, Shenzhen, 518033, China

**Keywords:** Sepsis, Cationic nanoparticle, Cell-free DNA, Reactive oxygen species, Macrophage polarization, Inflammation

## Abstract

Sepsis is a serious and life-threatening condition, which can lead to organ failure and death clinically. Abnormally increased cell-free DNA (cfDNA) and inflammatory cytokines are involved in the development and progression of sepsis. Thus, cfDNA clearance and down-regulation of inflammatory factors are essential for the effective treatment of sepsis. Here we designed and constructed a polydopamine-based multifunctional nanoparticle for the treatment of sepsis. These nanoparticles (NPs) are composed of polydopamine (PDA) grafted with cationic polyethyleneimine (PEI). On the one hand, the NPs can utilize the electrostatic interaction to effectively adsorb cfDNA in blood, then effectively inhibiting the activation of toll like receptors (TLRs) and nuclear factor kappa B (NF-κB) pathways induced by cfDNA. On the other hand, the NPs have an immunomodulatory function, which can effectively convert pro-inflammatory macrophage (M1) into anti-inflammatory macrophage (M2), thus reduce the release of inflammatory cytokines and slow down the inflammatory storm of sepsis. In addition, the NPs possess good reactive oxygen species (ROS) scavenging ability. Briefly, the effective treatment of sepsis can be achieved by multiple strategies of effectively capturing the inflammatory triggering factor cfDNA, modulating the polarization of M1 macrophage to M2 macrophage and scavenging ROS, which has a promising clinical application.

## Highlights

• Construction of multifunctional nanoparticle based on polydopamine.
• Scavenging ROS, capturing cell free DNA, and relieving inflammatory cytokine storm.
• Transforming macrophages from M1 to M2 and regulating inflammatory microenvironment.
• Reducing multiple organ damage and improving the survival rate of septic mice.
• Excellent anti-inflammatory ability and favorable biosafety.

## Graphical abstract

Sepsis is a serious and life-threatening condition that can arise from an infection, which can lead to organ failure and death in clinic. Abnormally increased cell-free DNA (cfDNA), massive accumulation of reactive oxygen species (ROS), increased pro-inflammatory macrophages and inflammatory cytokines are involved in the development and progression of sepsis. Thus, clearance of these inflammatory mediators and down-regulation of inflammatory factors are essential for the effective treatment of sepsis. Multifunctional nanoparticle with cfDNA scavenging, ROS scavenging and immunomodulatory functions can be effective in the treatment of sepsis and have good prospects for clinical application.

**Figure:**
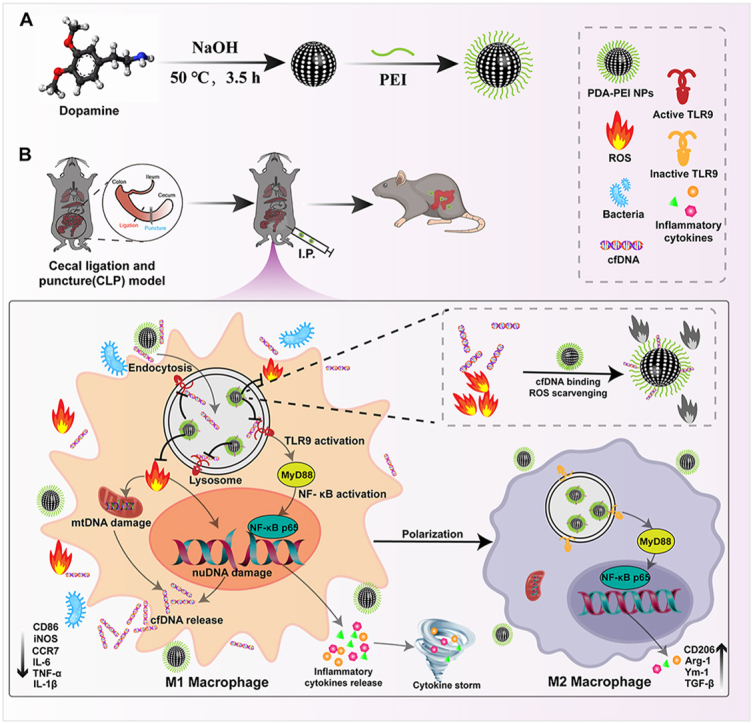

## 1 Introduction

Sepsis is the most common cause of mortality in hospital critical care units which caused 8 million deaths annually in worldwide [1,2]. Numerous clinical trials have failed due to the intricate nature of this disease [3]. Despite the best efforts of medical professionals, high morbidity and mortality are related to severe sepsis. The majority of approaches to managing these conditions have relied on supportive care, which involves providing intensive care and support to the patient's vital organs [4]. Sepsis is defined as an infection-related and persistent systemic inflammatory response syndrome (SIRS) with harmful effects, which causes endothelium, epithelium, and immune cells to be irreversibly damaged, ultimately leading to acute organ failure [5,6]. Sepsis leads to inflammatory dysregulation through the initiation of pattern recognition receptors (PRRs), especially Toll-like receptors (TLRs). With the progression of sepsis, TLRs and other PRRs play an important role in the abnormal inflammatory response, which contribute to sepsis-related high mortality [7]. Pathogen-associated molecular patterns (PAMPs) and endogenous danger-associated molecular patterns (DAMPs) are identified by PRRs, triggering immune responses [8,9]. cfDNA plays a significant role in sepsis, which includes CpG oligodeoxynucleotides from pathogens, endogenous DNA from neutrophil extracellular traps and damaged cells. The cfDNA triggers a sterile inflammatory response in sepsis [10]. Through identifying cfDNA, TLRs initiate intracellular signal cascades that leads to activate nuclear factor kappa B (NF-κB) and other transcription factors, leading to the release of inflammatory cytokines [11].

The activation of TLRs by cfDNA can be inhibited by the use of TLR antagonists or the removal of cfDNA. While various antagonists have been developed to treat inflammation-related diseases, their systemic inhibition of TLRs function can suppress immunity and increase infection risk [12,13]. The clearance of cfDNA can be accomplished by either degrading cfDNA or capturing cfDNA. DNase I is a nucleic acid endonuclease that can digest single-stranded or double-stranded DNA which is the most studied strategy for the degradation of cfDNA [14]. However, the protective effect of DNase I against sepsis is controversial, and it has been suggested that repeated injections of DNase I may aggravate the inflammatory response in sepsis [15]. cfDNA capturing is a strategy that utilizes negatively charged cfDNA combined with positively charged molecules to remove cfDNA. Cationic polymers have been investigated as a potential treatment for cfDNA-associated illnesses by scavenging cfDNA, but these polymers show dose-dependent toxicity [[16], [17], [18]]. With the application of nanotechnology in biotherapeutic field, more and more scholars are investigating the structural modification to construct more efficient and less toxic cationic nanosystems for cfDNA capture. Compared with cationic polymers, cationic nanomaterials have better inflammatory tissue targeting and retention, and are more efficient and safer cfDNA scavengers by improving cfDNA clearance and anti-inflammatory effects while reducing toxic side effects [19,20]. Cationic nanomaterials have achieved good therapeutic results in many inflammatory diseases, such as lupus, arthritis and colitis [[21], [22], [23], [24]]. Although capturing cfDNA can effectively inhibit the activation of the TLR inflammatory pathway, it does not completely resolve the organ damage caused by the inflammatory factor storm. M1 macrophages, as a kind of immune cells, can rapidly release a large amount of inflammatory factors to enhance the body's immune ability to achieve the killing of pathogens, but the production of excessive inflammatory factors also caused damage to normal tissue during sepsis [25]. Effective mitigation of the inflammatory storm as well as attenuation of organ damage involves decreasing the number of cells of the M1 phenotype while increasing the number of cells of the M2 phenotype [26,27].

**Scheme 1:**
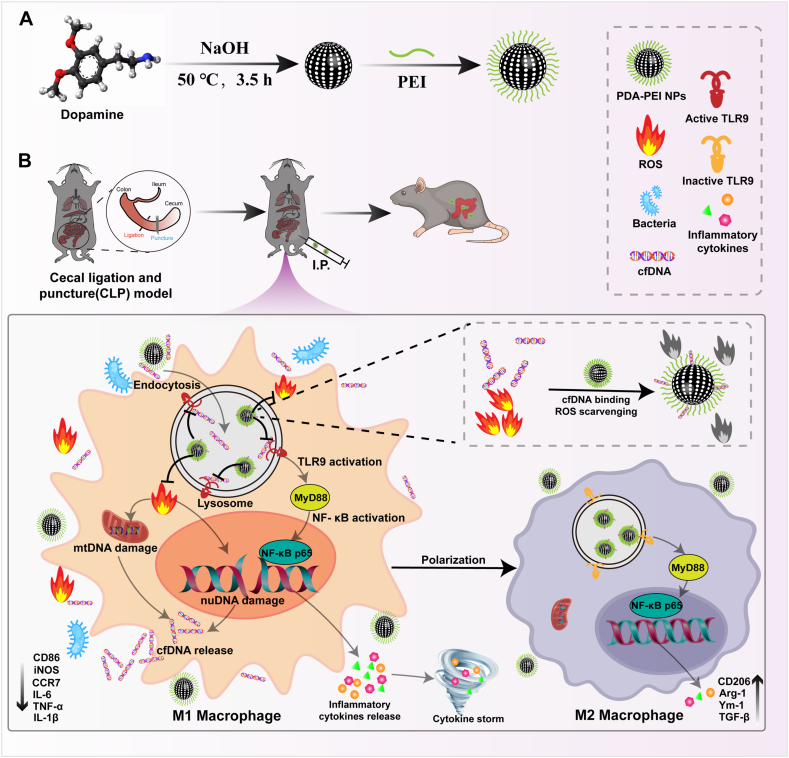
**The process by which PDA-PEI NPs suppress the inflammatory response and manage severe sepsis.** (A) Preparation of PDA-PEI NPs. (B) The mechanism of PDA-PEI NPs clearing cfDNA to inhibit pro-inflammatory responses and treat sepsis.

Considering the important role of cfDNA and M1 macrophages in the storm of inflammatory factors in sepsis, we propose a synergistic therapeutic strategy. Here, we have developed cationic nanoparticles PDA-PEI NPs for scavenging inflammatory cfDNA in circulation. This entails conjugating cationic polymer polyethyleneimine (PEI) onto polydopamine (PDA) through a simple one-pot process. This cfDNA scavenger exhibits strong binding affinity for cfDNA, efficiently inhibiting cfDNA-induced inflammation (Scheme 1). On the one hand, effective capture of cfDNA can be achieved by electrostatic interaction, then avoiding TLR activation. On the other hand, it can also polarize M1 macrophages with an inflammatory phenotype into M2 macrophages with an anti-inflammatory phenotype. In addition, the cationic nanomaterials can effectively remove inflammatory mediators such as reactive oxygen species, which is conducive to the self-repair of tissues and organs, thus reducing the level of inflammatory storms in the body by several methods, which is conducive to the valid treatment of sepsis.

## 2 Experimental section

### 2.1 Materials

Dopamine hydrochloride (DA), 3,3′,5,5′-Tetramethylbenzidine (TMB), NaOH and Copper sulfate pentahydrate (CuSO_4_·5H_2_O) were obtained from Aladdin (Shanghai, China). Polyethyleneimine (PEI, Mw = 10 kDa), xanthine oxidase, xanthine, 1,1-Diphenyl-2-picrylhydrazyl Free Radical (DPPH), and DiR iodide were obtained from Macklin (Shanghai, China). Fluorescein isothiocyanate (FITC) was purchased from MedChemExpress (USA). Lipopolysaccharides (LPS) from *Escherichia coli* 055: B5 was purchased from Biosharp (Hefei, China). Sangon Biotech (Shanghai, China) provided CpG 1826 and Cy5-CpG 1826. Gibco (USA) provided Fetal calf serum (FBS), Penicillin-Streptomycin, Roswell Park Memorial Institute 1640 (RPMI 1640), and Dulbecco's Modified Eagle's Medium (DMEM). Beyotime (Shanghai, China) provided the Calcein/PI Cell Viability/Cytotoxicity assay kit, mitochondrial membrane potential assay kit with 5,5′,6,6′-tetrachloro-1,1′,3,3′-tetraethylbenzimidazolylcarbocyanine iodide (JC-1), red blood cell lysis buffer, reactive oxygen species test kit, RIPA buffer, SDS-PAGE Sample Loading Buffer (5 × ), bovine serum albumin (BSA) and 4′,6-diamidino-2-phenylindole (DAPI). Meilunbio (Dalian, China) provided Cell Counting Kit-8 (CCK-8). Solarbio (China) provided Malondialdehyde (MDA) assay kit, paraformaldehyde and SuperRed/GelRed. DNA ladder Phosphate Buffered Saline (PBS), Tris-Glycine SDS-PAGE Running Buffer, Tris-Glycine Transfer Buffer, Tris Buffered Saline (TBS), TNF-α Mouse Uncoated ELISA, and IL-6 Mouse Uncoated ELISA were obtained from Servicebio (Wuhan, China). Aspartate aminotransferase (AST) assay kit, lactate dehydrogenase (LDH) assay kit, creatinine (Cre) assay kit, creatine kinase (CK) assay kit, alanine aminotransferase (ALT) assay kit, and urea (BUN) assay kit were obtained from Rayto Life and Analytical Sciences Institute (China). Qiagen (Hilden, Germany) provided DNA Blood Mini Kit. Invitrogen (California, USA) provided the Quant-iT PicoGreen dsDNA assay kit and QUANTI-Blue assay kit.

### 2.2 Synthesis of NPs

To be precise, dissolve dopamine hydrochloride (100.6 mg, 1 mmol) in 50 mL water, which was then heated to 50 °C with constant stirring. Then, 0.34 mL NaOH (sodium hydroxide, 1 mol/L, aq.) was gradually added to the solution. The reaction continued for 3.5 h. PDA NPs were then acquired using ultrafiltration tubes (MWCO = 50 kDa, Millipore, USA) by centrifugation at 4500 rpm, followed by washing three times. During the one-pot synthesis of PDA-PEI NPs. PEI (10 mg) was included in the mixture after 3.5 h, and two more hours were given to the response to proceed. PDA-PEI NPs were then produced by centrifugation at 4500 rpm using ultrafiltration tubes (MWCO = 50 kDa), which were then washed three times. The resultant PDA NPs and PDA-PEI NPs were lyophilized and then subjected to further characterization.

FITC-labeled NPs were created as follows. Fluorescein isothiocyanate (FITC) was covalently labeled at a consistence of 0.2 mg/mL on PDA NPs and PDA-PEI NPs (20 mg/mL) to create FITC-labeled NPs respectively. PDA-PEI NPs (20 mg/mL) and fluorescein isothiocyanate (0.2 mg/mL) were mixed for the covalent labeling procedure, and the mixture was agitated for a whole night. In order to remove any remaining free fluorescein, the FITC-labeled NPs were centrifuged and then thoroughly cleaned three times. Fluorescence microscopy was then used to trace these tagged nanoparticles.

### 2.3 Characterization

Many methods were used in the characterization of NPs. For the structures of these two types of nanoparticles, Fourier transform infrared spectroscopy (FTIR, Nicolet iS5, USA) and X-ray diffraction (XRD, MiniFlex 600, Japan) were used. These NPs' distribution and form were examined using transmission electron microscopy (TEM, Hitachi, Japan) and scanning electron microscopy (SEM, Hitachi, Japan). Zetasizer Nano ZEN (Malvern Panalytical, UK) was also used to measure the size, PDI and zeta potential of nanoparticles.

### 2.4 Cell culture

The American Type Culture Collection (ATCC, USA) provided the RAW264.7 cells and the InvivoGen (USA) provided HEK-Blue TLR9 reporter cell lines, which were then cultivated in DMEM added to 10 % FBS and 1 % Penicillin-Streptomycin solution. Every two days, cells underwent passage and the culture media was altered when they reached 85 %-90 % confluence. The cells were then gathered for additional study.

### 2.5 Animals

The Animal Ethical and Welfare Committee of Sun Yat-sen University has authorized all animal studies. Male C57BL/6 mice, aged six to eight weeks, were obtained from the Laboratory Animal Center of SYSU, situated in Guangzhou, China.

### 2.6 DNA binding capacity

To assess the ability of DNA binding, Quant-iT PicoGreen concentrate (12.5 μL) and 2.5 mg/mL CpG 1826 (20 μL) were combined in 10 mL TE buffer and TE buffer with 10 % FBS respectively. The samples of varying concentrations (treated samples group) or Milli-Q water (blank group) in 100 μL were added to a 96-well plate along with 100 μL of the mixed solution, followed by incubation 30 min at 37 °C. Next, the PicoGreen-DNA complex's fluorescence intensity was determined with a microplate reader (λ_ex_ = 490 nm and λ_em_ = 520 nm). The DNA binding affinity was calculated using the provided formula, and all operations were conducted in the dark.

NPs were also found to have the ability to bind DNA by agarose gel electrophoresis using CpG 1826 as the template. Before the trials, PBS was used to solubilize DNA and disperse NPs. After 15 s of vortexing, 10 mL of the NPs solution (1 mg/mL) and 1 mL of the DNA solution (0.2 mg/mL) were combined, and incubating the mixture for 30 min. Then 100 μL of complex suspensions were run on agarose gel (1 %) at 100 V for 40 min at 37 °C.

### 2.7 Toxicity assay

#### 2.7.1 CCK-8 test

Using the CCK-8 test to assess PDA-PEI NPs' cytotoxicity as follows: In 96-well plates, planting RAW264.7 cells of 5000 cells per well. Wait until the cells adhere to the wall, the growth medium was supplemented with PDA NPs, PEI and PDA-PEI NPs at several concentrations (0, 1, 5, 10, 20, 50, 100, and 200 μg/mL). Following incubation for 1 and 2 days respectively, PBS was used to wash the cells three times before being re-incubated 1 h in 100 μL volume of medium contain 10 % CCK-8. Microplate reader was used to measure the absorbance at 450 nm, and cell viability was determined by comparing the outcomes with those of the control group, which received only fresh media. Three duplicates of the experiment were conducted.

#### 2.7.2 Assay for live/dead staining

To test cell viability, the CAM/PI Cell Viability/Cytotoxicity Assay Kit was used. In a 6-well plate, a density of 1 × 10^6^ RAW264.7 cells was planted into each well and cultivated for an entire night. After that, the cells were subjected to various dosages of PDA-PEI NPs (0, 1, 5, 10, 20, 50, 100, and 200 μg/mL) for a duration of 24 h. Then CAM (λ_ex_ = 494 nm and λ_em_ = 517 nm) and PI (λ_ex_ = 535 nm and λ_em_ = 617 nm) were incubated in the well plate for 30 min in the absence of light. Using fluorescence microscope (DMi8-M, Leica, Germany) to take pictures.

#### 2.7.3 Biocompatibility of NPs *in vivo*

Each of the three groups of five healthy mice was allocated at random: the saline group, the PDA group, the PEI group and the PDA-PEI group. At 0 h and 24 h, the mice were given intraperitoneal injections of NPs at a dosage twice as therapeutic (20 mg/kg of NPs). After 24 h following the final injection, blood was extracted from the eyeballs in order to calculate the biochemical indices (ALT, AST, BUN, Cre, CK, and LDH) and the number of blood cells (RBC, PLT and NEUT). After drawing blood, euthanize these mice, and take out their heart, liver, spleen, lung, kidney, and intestine. HE staining allowed for the observation of organ disease.

### 2.8 Mitochondrial membrane potential assay

Using JC-1 dye to evaluate mitochondrial membrane potential. RAW264.7 cells were seeded with cells from each well at a density of 5 × 10^5^ and placed in a 6-well plate. PDA NPs (50 μg/mL) and PDA-PEI NPs (50 μg/mL) were added to the pores for pretreatment for 1 h. Subsequently, the cells were stimulated with H_2_O_2_ (400 μM) for 24 h. Then stain with JC-1 at 37 °C for 20 min and take photos using fluorescence microscope (DMI8, Leica, Germany).

### 2.9 Hemolysis assay

Purified red blood cells were acquired by blood sample centrifugation for 5 min at 3000 rpm. Then wash three times with sterile saline. After that, different PDA-PEI NPs solution concentrations were combined with the red blood cells to produce a 2 % erythrocyte suspension (v/v). The mixtures were vortexed and then cultivated 3 h at 37 °C. Following the supernatants were collected and centrifuged, the absorbance (Abs) at 540 nm wavenumber was measured. PBS (pH 7.4) and ultrapure water were used to treat erythrocytes as both positive and negative controls. Hemolysis (%) formula used to calculate the hemolysis rate.

(1) $$Hemolysis\;\left(\%\right)=\frac{\left(Abs\;of\;sample-Abs\;of\;negative\;control\right)}{\left(Abs\;of\;positive\;control-Abs\;of\;negative\;control\right)}\times 100\;\%$$

### 2.10 Colocalization of cfDNA and NPs

This experiment aimed to investigate the intracellular binding capability of synthesized PDA-PEI NPs to cfDNA. Cy5-labeled CpG 1826 served as a typical cfDNA. Initially, RAW264.7 cells were inoculated in a confocal dish at a density of 1.5 × 10^5^ cells per well and cultured in Cy5-CpG (1 μM) for 4 h. FITC-labeled NPs (1 mg/mL) were included into the medium after excess CpG was removed. Following an 8 h incubation period, DAPI staining was applied to the treated cells for confocal microscopy observation (LSM880, Zeiss, Germany).

### 2.11 ROS scavenging

The oxidation of TMB was used to measure the scavenging efficacy of PDA-PEI NPs on ·OH radicals. CuSO_4_·5H_2_O and H_2_O_2_ underwent a Fenton reaction to produce hydroxyl radicals. CuSO_4_·5H_2_O (2 mM), H_2_O_2_ (5 mM), and NPs (10-200 μg/mL) was blended together, then incubated at room temperature for 1 h. By calculating the distinctive absorbance at 650 nm of oxidized TMB (1 mM), the amount of hydroxyl radicals eliminated was ascertained.

Similar to earlier research, PDA-PEI NPs' ability to scavenge free radicals was evaluated. First, a 95 % ethanol solution containing 0.1 mM DPPH was made. Subsequently, different dosages of PDA-PEI NPs or ascorbic acid, varying from 10 to 200 μg/mL, with 4 mL of DPPH solution were mixed. By tracking the drop in absorbance at 516 nm, the scavenging activity was evaluated after 30 min in the dark.

To observe the intracellular ROS clearance ability of NPs, RAW264.7 cells were put onto 6-well plates 5 × 10^5^ cells per well, and they were left to adhere for the whole night in growth medium. Subsequently, the cells were subjected to 6 h of exposure to different concentrations of NPs and 1 μg/mL of LPS. Following three PBS washes, DCFH-DA probe was accustomed to stain the cells, then there were further PBS washes, the pictures was captured with fluorescent microscope (DMi8-M, Leica, Germany).

Furthermore, flow cytometry was employed to ascertain the percentage of RAW264.7 cells displaying a high ROS level. The cells underwent the previously specified treatment conditions, and CytoFLEX LX (Beckman, USA) was used for analysis.

### 2.12 Malondialdehyde (MDA) content determination

To evaluate lipid peroxidation level in the RAW264.7 cells, RAW264.7 cells were put onto 6-well plates 5 × 10^5^ cells per well, and they were left to adhere for the whole night in growth medium. After 2 h treatment of LPS (1 μg/mL), the cells were subjected to 6 h of exposure to NPs (50 μg/mL), then Malondialdehyde (MDA) assay kit was used to detect intracellular MDA content.

### 2.13 In vitro anti-inflammatory assays

In each well of a 96-well plate, 2 × 10^4^ RAW264.7 cells were planted. Following a 30 min incubation period, 2 μL of CpG 1826 (1 mg/mL) was included to the cells. After 20 min, PDA-PEI NPs were introduced in a 200 μL final volume at a concentration of 50 μg/mL. Following a further 24 h incubation period, supernatants were extracted and evaluated utilizing an ELISA kit for Tumor Necrosis Factor-α (TNF-α).

In order to examine alterations of inflammatory cytokines in RAW264.7 cells subsequent to PDA-PEI NPs (50 μg/mL) treatment, immunofluorescent labeling was utilized to detect TNF-α protein expression. Cells were first fixed in a 4 % paraformaldehyde solution for 10 min. Following a 15 min treatment with 3 % H_2_O_2_, goat serum was used to inhibit the cells in order to prevent nonspecific binding. Subsequently, they were treated individually with the TNF-α primary antibody (1:200, Abcam, USA, ab183218) for an entire night at 4 °C. After 1 h in the dark with FITC-anti-rabbit IgG (1:100, Servicebio, China), cells were labeled with DAPI for 5 min. The pictures were captured using fluorescence microscope (DMI8, Leica, Germany).

### 2.14 In vitro TLR9 activation assay

HEK-Blue TLR9 reporter cells were planted at a density of 5 × 10^4^ per well in a 96-well plate. Then, cells were incubated with CpG 1826 (1 μg/mL) for 30 min, and PDA-PEI NPs (50 μg/mL) were added to incubate for 24 h, the supernatants were collected and incubated with QUANTI-Blue. The corresponding embryonic alkaline phosphatase (SEAP) activity in each well was determined by measuring the optical density at 620 nm (OD_620 nm_) using a microplate reader.

### 2.15 CLP-induced sepsis model

In accordance with recognized protocols, mice were given the cecal ligation and puncture (CLP) procedure to cause sepsis [28]. In short, mice were shorn of their abdomen hair and given a 50 mg/kg dose of pentobarbital to anesthetize. The cecum was made visible by making a 1 cm incision in the abdomen's midline. A 21-gauge needle and a 4-0 silk ligature were used to make two perforations in the distal 50 % of the cecum. After that, 4-0 silk suture was used to close the incision on the abdomen, and a small quantity of feces was inserted back into the abdominal cavity together with the cecum. For fluid resuscitation, 1 mL of sterile saline was injected subcutaneously. Using an electric blanket to maintain the mice's body temperature between 36.0 °C and 37.5 °C for the entire process.

### 2.16 Treatment of mice with sepsis

Ten mice per group were used for the distribution of the control, sham, CLP, PDA, and PDA-PEI groups. As previously described, the CLP procedure was carried out on mice without receiving any additional care in the CLP group. The mice in the sham group had an abdominal laparotomy as the sole process, with no further CLP. PDA NPs and PDA-PEI NPs were given to the PDA and PDA-PEI groups 1 h and 12 h following CLP at 10 mg/kg. Using a previously protocol, body weight, survival rates, and clinical assessments were tracked for seven days in a row [29]. Clinical scores had the following definitions: 0, absent symptoms; 1, huddling and piloerection; 2, huddling, piloerection, and severe diarrhea; 3, absence of curiosity in the environment and extreme diarrhea; 4, reduced mobility and dull look; 5, lack of the ability to correct oneself. When the clinical score of mice hit 5, it was time to put it to sleep. Serum was taken 24 h following CLP in order to quantify the quantities of cfDNA, Interleukin-6 (IL-6), and Tumor Necrosis Factor-α (TNF-α).

### 2.17 Histopathological analyses

The murine blood was extracted for biochemical analysis after different treatments. The heart, lung, kidney, spleen, intestine, and liver tissues of mice were fixed in 4 % paraformaldehyde for a whole day, then ethanol was used to dry the tissues and xylene was used to permeabilize them. Tissues were then sectioned into 5 μm slices and fixed in paraffin for further use. The slices underwent stained using hematoxylin and eosin (H&E) following dewaxing and dehydration. Images were taken and examined through the use of a microscope (VS200, Olympus, Japan).

### 2.18 Biodistribution

Four groups of C57BL/6 mice, aged six to eight weeks, were randomized: sham + saline, sham + DiR-labeled PDA-PEI NPs, CLP + saline and CLP + DiR-labeled PDA-PEI NPs. Mice were intraperitoneally injected with DiR-labeled PDA-PEI NPs that had been dissolved in 100 μL of sterile saline (equivalent to 0.2 mg/kg DiR), CLP group were administered 1 h after surgery. The control group received the equal amount of saline. In the following order, the mice were put to death 2 h, 12 h, and 24 h after CLP. The fluorescence intensity of the lung, kidney, intestine, liver, spleen, and heart was measured utilizing an *in vivo* imaging apparatus for small animals (IVIS Lumina III, USA). The organs underwent fluorescence quantitative analysis.

### 2.19 cfDNA extraction and quantitation

The QIAamp DNA Blood Mini Kit was utilized to extract cfDNA from the serum and peritoneal lavage fluid of mice. The Quant-iT PicoGreen dsDNA Assay Kit was utilized to measure the quantity of cfDNA.

### 2.20 Isolation of peritoneal macrophages

The isolation of peritoneal macrophages was done as previously [30]. In short, inject 3 mL of precooled PBS into the abdominal cavity of mice for lavage. Suck out the peritoneal lavage fluid, lyse the red blood cells with the red blood cell lysis fluid, and resuspend the cells in a 60 mm culture dish with RPMI 1640 containing 10 % fetal bovine serum in an incubator with 5 % CO_2_. Adherent cells were cultivated further after non-adherent cells were eliminated.

### 2.21 Western blot assay

To extract proteins, RIPA buffer was added to cultured cells 30 min on ice. Collect cell suspension and centrifuge at 12000 rpm (4 °C) for 15 min, then take the supernatant. Next, the BCA test (KeyGentec, China) was used to ascertain the protein concentration. Equal volumes of protein samples were then combined with loading buffer (5 × ), and the mixture was heated at a temperature of 100 °C for 5 min 10 % sodium dodecyl sulfate-polyacrylamide gel electrophoresis (SDS-PAGE) was then accustomed to divide the samples. Following protein separation, the membranes made of polyvinyl difluoride (Millipore, USA) were blocked for 1 h using 5 % BSA. The protein bands were then incubated overnight at 4 °C with primary antibodies against NF-κB p65 (1:1000), Phospho NF-κB p65 (1:1000), MyD88 (1:1000), TLR9 (1:500), iNOS (1:1000), Arg-1 (1:1000), CD86 (1:1000), CD206 (1:1000), CCR7 (1:1000), Ym-1 (1:1000), and GAPDH (1:3000). After then, the secondary antibody was incubated for an extra hour at 37 °C. The Omni-ECLTM Femto Light Chemiluminescence Kit (Vazyme, China) and imaging system (Bio-Rad, USA) was used to view the proteins. The protein expression level analysis was standardized to GAPDH using Image J software.

### 2.22 Real-time quantitative polymerase chain reaction analysis

RNA was extracted from cells through trizol reagent (Invitrogen, USA). Then utilizing the Evo M-MLV RT Premix for q-PCR (Accurate Biotechnology, China) and the All-in-OneTM miRNA First-Strand cDNA Synthesis Kit (GeneCopoeia, USA), cDNA was created by reverse transcribed RNA. mRNA was subjected to qRT-PCR using the SYBR® Green Premix Pro Taq HS qPCR Kit (Accurate Biotechnology, China). Table S2 contains the primer sequences created for this investigation. The 2^−^^ΔΔCT^ technique was utilized to quantify the amplified transcripts.

### 2.23 Enzyme linked immunosorbent assay

Murine blood, cell culture supernatant, and peritoneal lavage fluid were centrifuged for 10 min at 3000 rpm, then kept at 4 °C. ELISA kits were employed in compliance with the manufacturer's recommendations to test the levels of TNF-α and IL-6 cytokines.

### 2.24 Macrophage polarization was analyzed by flow cytometry

In order to evaluate the effect of PDA-PEI NPs on macrophage polarization, the expression of CD86 and CD206 in peritoneal macrophages was detected by flow cytometry. Cells were stimulated with LPS (1 μg/mL) for 2 h, then added to NPs, and 12 h later, 1 × 10^6^ cells were collected. The Fc receptor was first blocked with using TruStainFcX™ PLUS. Then the cells were incubated with 100 μL PBS containing CD86/PE antibody at 4 °C and CD206/Alexa Fluor 647 antibody for 30 min at 4 °C. Flow cytometry analysis was performed.

### 2.25 Immunofluorescence

The lung tissue sections from mice were deparaffinized, and then the antigen was detected using an EDTA antigen retrieval buffer (pH 8.0). To inhibit endogenous peroxidase activity, slices were treated for 25 min in an aqueous solution containing 3 % hydrogen peroxide without any light. Then they were inhibited for 30 min with 3 % BSA. After that, sections were treated with primary antibodies against TNF-α (1:100), Arg-1 (1:100), iNOS (1:100), and IL-6 (1:100) overnight at 4 °C. The secondary antibody was then applied to the sections for 50 min at 37 °C. Additional staining for iNOS and Arg-1 was carried out for 10 min at room temperature without light using iF647-Tyramide Signal Amplification (1:500, Servicebio, China) and FITC-Tyramide Signal Amplification (1:500, Servicebio, China), respectively. Primary antibodies against F4/80 (1:200) were used to treat sections containing iNOS and Arg-1. Then, fluorescent secondary antibodies labeled with CY3 (1:100, Servicebio, China) was added. Similar protocols using CY3-labeled fluorescent secondary antibodies were applied to sections containing IL-6 and TNF-α. DAPI was used to stain the nuclei. Pictures were taken using a slide scanner (VS200, Olympus, Japan). The antibodies used in this study are listed in Table S1.

### 2.26 Statistical analysis

For statistical analysis, GraphPad Prism software 9 (La Jolla, CA) was utilized, and the findings were shown as mean ± SD. For straightforward two-sample comparisons, the student's t-test was employed to assess variations across groups, (∗*p* < 0.05, ∗∗*p* < 0.01, ∗∗∗*p* < 0.001, ∗∗∗∗*p* < 0.0001). For multiple comparisons, One-way analysis of variance was used with Tukey's post hoc test (ANOVA). We used the Kaplan-Meier method to contrast variations in survival rates.

## 3 Results

### 3.1 Characterization of PDA-PEI NPs

**Fig. 1:**
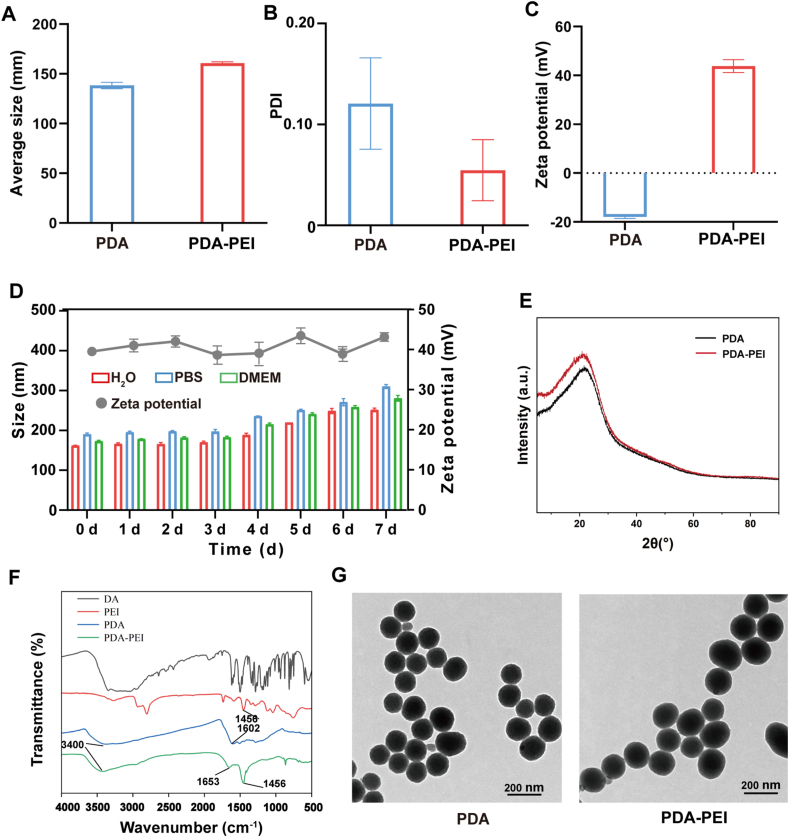
Characterization of NPs. (A) The hydrodynamic size of PDA NPs and PDA-PEI NPs. (B) The PDI of PDA NPs and PDA-PEI NPs. (C) The zeta potential of PDA NPs and PDA-PEI NPs. (D) The particle size and zeta potential of the PDA-PEI NPs for 7 days. n = 3. (E) PDA NPs and PDA-PEI NPs' XRD spectra. (F) PDA NPs and PDA-PEI NPs' FTIR spectra. (G) TEM picture of PDA NPs and PDA-PEI NPs. Scale bar = 200 nm.

A simple one-pot method was used to synthetize PDA-PEI NPs. First, dopamine self-polymerized to create polydopamine (PDA) in an alkaline pH environment. Then, in the same alkaline pH range, polydopamine interacted with amino groups in polyethyleneimine (PEI, Mw = 10 kDa). Fig. 1A-C displays the NPs' hydrodynamic size, polydispersity index (PDI) and zeta potential. PDA-PEI NPs showed a hydrodynamic size of 161 ± 6 nm and PDI of 0.07, which is similar to PDA NPs. After modification of PDA NPs with PEI, the zeta potential of NPs increased from −18.0 mV to 43.8 mV. The nanoparticles were then incubated for 7 days in H_2_O, PBS, and DMEM containing 10 % FBS, stability analysis showed that the particle size remained stable for 7 days. Meanwhile, the zeta potential of nanoparticles in H_2_O remained stable for 7 days (Fig. 1D). The absence of a crystal structure in the NPs was shown by X-ray diffraction (XRD) spectra (Fig. 1E). FTIR spectra were employed to confirm the structure of PDA-PEI NPs (Fig. 1F). The characteristic adsorption band of PDA-PEI at nearly 3400 cm^−^^1^ was ascribed to the stretching vibration of -OH, phenolic O-H and active N-H. The peak at 1602 cm^−^^1^ was due to the stretching vibrations of O-H or C=O stretching from catechol groups of PDA. In the meantime, for PDA-PEI, the characteristic peak shift to 1653 cm^−^^1^, this might be an overlap of multiple peaks such as N-H bending vibration, C=O stretching vibration, and C=N stretching vibration. For PEI and PDA-PEI, the peak of 1456 cm^−^^1^ corresponded to the N-H bending mixed with CH_2_. The above results indicating the successful modification of PEI onto PDA. PDA NPs and PDA-PEI NPs both had spherical forms with sizes smaller than 200 nm, according to pictures of the NPs obtained using TEM and SEM (Fig. 1G and Fig. S1).

In conclusion, PDA-PEI NPs' shape, chemical makeup, and crystal structure were very similar to those of PDA NPs. It is implied by this that the addition of polyethyleneimine groups changed the positive charge density but didn't change the nanostructures traits of PDA NPs.

### 3.2 Cytotoxicity and cytoprotective capacity

**Fig. 2:**
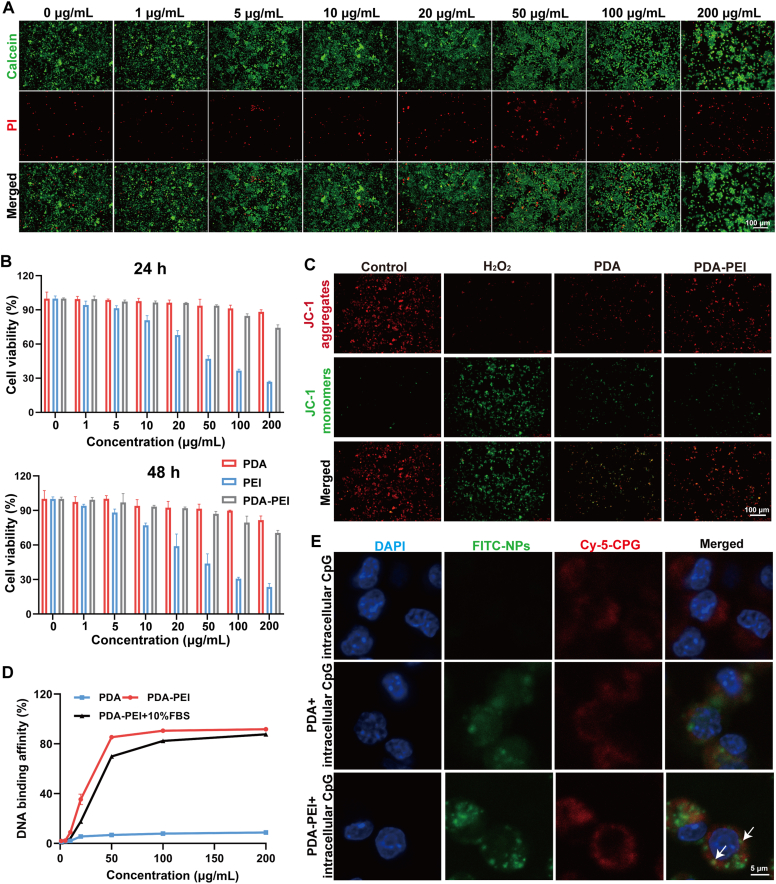
Cytotoxicity, cytoprotective capacity, DNA binding affinity and colocalization of NPs. (A) Live/dead staining of PDA-PEI NPs utilized for RAW264.7 cells for 24 h. Scale bar = 100 μm. (B) The CCK-8 test was accustomed to measure the cytotoxicity of PDA NPs, PEI, and PDA-PEI NPs in RAW264.7 cells for 24 h and 48 h. n = 3. (C) Fluorescence images of JC-1-stained RAW264.7 cells after different treatments. Scale bar = 100 μm. (D) Affinity of PDA NPs and PDA-PEI NPs for binding DNA in TE buffer and TE buffer with 10 % FBS. n = 3. (E) The colocalization of CpG and NPs in RAW264.7 cells following 8 h of cultivation. As indicated with white arrows, co-localization of cfDNA and PDA-PEI NPs showed up as yellow spots. Scale bar = 5 μm.

We assessed the toxicity of PDA-PEI NPs against cells using the live/dead staining test and the CCK-8 test. The live/dead staining test in Fig. 2A demonstrated cell death was slightly increased when the concentration of PDA-PEI NPs below 50 μg/mL, while significantly increased when the concentration of PDA-PEI NPs above 100 μg/mL. The quantified outcomes of live/dead staining was shown in Fig. S2. We then compared the cell viability of PDA NPs, PEI, PDA-PEI NPs at different concentrations by CCK-8 experiment, the results indicated that PDA-PEI NPs did not affect the cell viability of RAW264.7 cells at 24 h and 48 h within 0-50 μg/mL concentration, the cell viability decreased significantly when the PEI concentration was more than 10 μg/mL. In addition, cell viability began to decline when PDA-PEI NPs concentrations were higher than 100 μg/mL, but remained higher than PEI (Fig. 2B). Consequently, we selected 50 μg/mL of PDA-PEI NPs for further research. Additionally, the hemolytic abilities of PDA-PEI NPs were assessed (Fig. S3). The NPs did not show signs of hemolysis compared with the positive control. Furthermore, the protective efficacy of PDA-PEI NPs was investigated by live/dead staining and 5,5′,6,6′-tetrachloro-1,1′,3,3′-tetraethylbenzimidazolylcarbocyanine iodide (JC-1). Comparing the LPS-induced cells control group with the normal group, there was a discernible raise in dead cells and a substantial drop in the number of live cells. Conversely, treatment with PDA-PEI NPs (50 μg/mL) increased the percentage of live cells while decreasing the percentage of dead cells (Figs. S4A and B). This indicates that at 50 μg/mL, PDA-PEI NPs showed negligible cytotoxicity and had a protective effect against LPS-induced cell inflammation. Mitochondria play an important role in regulating cell apoptosis, and the decrease in mitochondrial membrane potential (MMP) is widely considered a marker of cell apoptosis and death. To evaluate the effect of PDA-PEI NPs on MMP, RAW264.7 cells were exposed to PDA-PEI NPs and subsequently stained with JC-1. JC-1 accumulates and forms JC-1 aggregates in the mitochondrial matrix of cells with high MMP, producing strong red fluorescence. After hydrogen peroxide treatment, the red fluorescence decreased while the green fluorescence increased, indicating a decrease in MMP content. However, the addition of PDA-PEI NPs significantly weakened the increase in green fluorescence, indicating that PDA-PEI NPs prevented the reduction of MMP and inhibited cell apoptosis (Fig. 2C).

### 3.3 DNA binding affinity and colocalization

cfDNA is a critical biomarker for sepsis prognosis and prediction, and there is a direct relationship between sepsis patient mortality and cfDNA concentration [10]. Intracellular cascade signaling and the release of inflammatory cytokines occur when toll-like receptors (TLRs) bind to cfDNA [31]. Thus, it is anticipated that eliminating cfDNA or obstructing aberrant cfDNA pathways will reduce systemic inflammation in sepsis, lessen organ damage, and result in successful treatment. We first assessed the DNA binding affinity of PDA-PEI NPs in the study using PicoGreen DNA quantification kit. By monitoring the variation in PicoGreen-labeled DNA fluorescence intensity, the DNA affinity efficiency of NPs was ascertained. The outcomes showed that PDA-PEI NPs had outstanding DNA binding ability, the presence of 10 % FBS reduced DNA binding at a low NPs concentration, possibly due to FBS proteins competitively binding the DNA or the NPs, but DNA binding was restored at higher NPs concentration (Fig. 2D). This result implies that PDA-PEI NPs could capture cfDNA efficiently, providing a foundation for further studies on their ability to reduce systemic inflammation by capturing cfDNA in the treatment of sepsis. Similar results were observed through agarose gel electrophoresis (Fig. S5), the bands of PDA were similar to those of free CpG 1826, indicating that there was no loss of DNA after PDA treatment, and that PDA had little DNA binding ability. However, the bands of PDA-PEI NPs were weakened, which indicated that PDA-PEI NPs could effectively interact with negatively charged DNA and prevent DNA migration in gel electrophoresis.

Next, we examined CpG and NPs colocalization in RAW264.7 cells (Fig. 2E). The confocal colocalization ratio of PDA-PEI NPs (FITC-labeled, green) with CpG (Cy5-labeled, red) is more than PDA NPs, suggesting that PDA NPs coated with PEI possess a significant binding capacity to cfDNA. Consistent results were obtained from quantitative analysis using FCM. The difference in fluorescence intensity between PDA NPs and PDA-PEI NPs at 8 h and 12 h was significantly different (Fig. S6), this may be due to the fact that positively charged nanoparticles are more likely to bind to negatively charged cell membranes and be taken up by cells.

### 3.4 ROS scavenging and anti-inflammatory effect

**Fig. 3:**
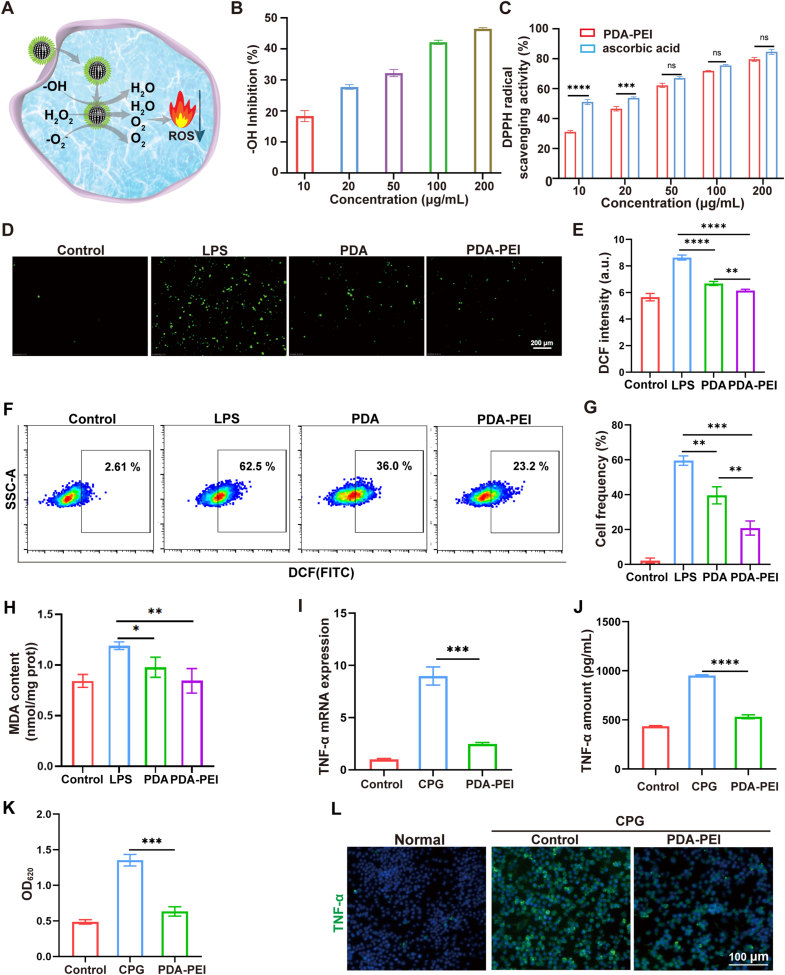
NPs possess ROS scavenging, anti-inflammatory and macrophage polarizing abilities. (A) The mechanism of PDA-PEI NPs to scavenge ROS. (B) The ability of PDA-PEI NPs to scavenge hydroxyl radicals (·OH). n = 3. (C) The ability of PDA-PEI NPs and ascorbic acid to scavenge DPPH radicals. n = 3. (D) RAW264.7 cells intracellular ROS imaging after treatment. Scale bar = 200 μm. (E) Image J quantifies the fluorescence intensity of the pictures in Fig. 3D. n = 3. (F) Assessment of RAW264.7 cells intracellular ROS by flow cytometry after treatment. (G) Corresponding quantitative analysis of Fig. 3F. (H) Quantification of MDA content in RAW264.7 cells subjected to different treatments. n = 3. (I) TNF-α mRNA expression. n = 3. (J) TNF-α cytokine level in cellular supernatant. n = 3. (K) Activation of HEK-TLR9 reporter cells by CpG in the absence or presence of PDA-PEI NPs. The SEAP activity in supernatants was determined with a QUANTI-Blue assay at OD_620 nm_. n = 3. (L) Immunofluorescence of TNF-α. (*∗p* < 0.05, *∗∗p* < 0.01, *∗∗∗p* < 0.001, *∗∗∗∗p* < 0.0001). (For interpretation of the references to color in this figure legend, the reader is referred to the Web version of this article.)

ROS play a critical role in sepsis-induced inflammation [32]. The Removal of ROS can significantly prolong the survival time of sepsis mice [33]. PDA is a naturally occurring antioxidant that can be used to scavenge ROS [34,35]. The mechanism of PDA-PEI NPs to scavenge ROS is shown in Fig. 3A, ROS clearance efficiency of PDA-PEI NPs was assessed through an examination of the removal of hydroxyl radicals (·OH) and DPPH radicals [36,37]. As shown in Fig. 3B, we evaluated the ability NPs to scavenge ·OH produced through the Fenton reaction of Cu^2+^ and H_2_O_2_. To assess the ·OH scavenging capacity of NPs, the change in the absorbance kinetics of the TMB product that was oxidized by ·OH at 650 nm was examined. As the concentration of PDA-PEI NPs raised, the efficiency of hydroxyl radical inhibition also increased. Next, we used the DPPH assay to measure the antioxidant potential of the sample *in vitro*. Alcoholic solutions of DPPH have a maximum absorption at 517 nm, and are a stable free radical that can be stabilized in organic solvents. The one electron of DPPH is taken up by free radical scavengers, causing its color to lighten. Evaluation of the antioxidant capacity of NPs by the change in absorbance at the wavelength of maximum light absorption. As the concentration of nanoparticles and ascorbic acid increases, the ability to eliminate free radicals also increases. After reaching a 50 μg/mL concentration, PDA-PEI NPs and ascorbic acid, a traditional antioxidant, did not vary in the capacity to eliminate free radicals (Fig. 3C). Then, cell-permeant dichlorodihydrofluorescein diacetate (DCFH-DA) was utilized as a sign to evaluate the intracellular ROS level. LPS-treated cells showed bright green fluorescence (Fig. 3D and E), indicating that oxidative pressure had been successfully induced. The fluorescence intensity in the cells dramatically dropped when PDA-PEI NPs were added, suggesting that the NPs scavenged ROS *in vitro*. Flow cytometry of the cells receiving various treatments stained with the ROS fluorescent probe DCFH-DA further supported this (Fig. 3F and G). In addition, we have assessed RAW264.7 cells intracellular ROS by flow cytometry after different doses of nanoparticles treatment (20 μg/mL, 50 μg/mL, 100 μg/mL) and found that PDA-PEI NPs cleared ROS in a dose-dependent manner (Figs. S7 and S8). Malondialdehyde (MDA) is a product of membrane lipid peroxidation and one of the important indicators of oxidative stress, as shown in Fig. 3H, the content of MDA in cells increased under the stimulation of LPS, and decreased after NPs treatment, indicating that the oxidative stress of cells was alleviated.

Previous research proposed that inflammatory circulating cfDNA activates TLR9 is crucial for sepsis development [[38], [39], [40]]. Here we utilized CpG 1826 (1 μg/mL) to activate TLR9 in RAW264.7 cells and assess anti-inflammatory properties of PDA-PEI NPs. As depicted in Fig. 3I and J, TNF-α transcription and translation were decreased by PDA-PEI NPs administration, suggesting that NPs prevent the inflammation brought on by nucleic acids *in vitro*. Then we used the HEK-TLR9 reporter cells, and found that PDA-PEI NPs significantly inhibited CpG-induced TLR9 activation (Fig. 3K). In addition, the immunofluorescence results also showed CpG 1826 inducement notable enhanced the TNF-α expression by macrophage, and this increase was mitigated by PDA-PEI NPs treatment (Fig. 3L).

### 3.5 Phenotypic shift from M1 to M2 in macrophages

**Fig. 4:**
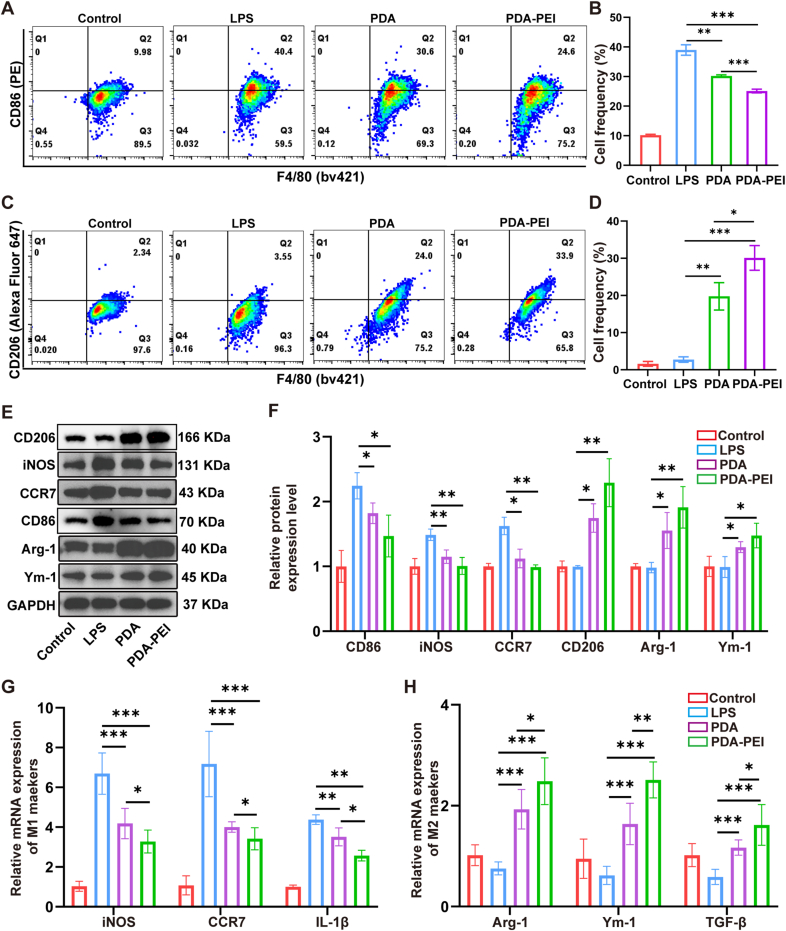
NPs switches M1-type macrophages to M2-type macrophages *in vitro*. (A) Flow cytometry results showed that NPs could reduce M1-type macrophages. (B) Corresponding quantitative analysis of Fig. 4A. n = 3. (C) Flow cytometry results showed that NPs could increase M2-type macrophages. (D) Corresponding quantitative analysis of Fig. 4C. n = 3. (E) Protein expression was assessed by western blot of M1 indicators (CD86, CCR7, iNOS) and M2 indicators (CD206, Arg-1, Ym-1). (F) Quantitative results for western blot in Fig. 4E. n = 3. (G and H) The mRNA expression of M1 indicators (iNOS, CCR7, IL-1β) and M2 indicators (Arg-1, Ym-1, TGF-β) was determined. n = 6. (∗*p* < 0.05, ∗∗*p* < 0.01, ∗∗∗*p* < 0.001).

Macrophages can be activated into two distinct phenotypes: pro-inflammatory M1 and anti-inflammatory M2 [41]. M1 macrophages primarily contribute to the activation of different inflammations by secreting mediator of inflammation. M2 macrophages have a significant anti-inflammatory effect and are essential for tissue homeostasis restoration and wound healing [42,43]. The conversion of M1 macrophages to M2 macrophages is considered a possible therapy for sepsis [44,45]. According to previous studies, ROS are related to the regulation of macrophage phenotypes, and that scavenging ROS promotes the conversion of M1 macrophages into M2 macrophages [46]. The above results show that PDA-PEI NPs have a significant capacity to scavenge ROS. To assess the potential of PDA-PEI NPs for macrophage reprogramming, peritoneal macrophages were subjected to a 24 h LPS (1 μg/mL) incubation to establish the M1 polarization phenotype. The M1 macrophages were then treated with NPs for a duration of 12 h. Flow cytometry analysis of the CD86 (biomarker of M1-type macrophages) and CD206 (biomarker of M2-type macrophages) showed that the number of M1-type macrophages was significantly reduced (Fig. 4A and B) and the number of M2-type macrophages was significantly increased (Fig. 4C and D) after incubation with PDA NPs and PDA-PEI NPs compared to the LPS-treated group. As well as western blot assay, in both the PDA NPs and PDA-PEI NPs groups, M1 indicators (CD86, iNOS and CCR7) protein expression sharply downregulated, whereas M2 indicators (CD206, Arg-1 and Ym-1) protein expression upregulated (Fig. 4E and F). Similar results were observed with qRT-PCR, after NPs were added, M1 indicators (CCR7, IL-1β and iNOS) mRNA expression was notably downregulated, whereas M2 indicators (Arg-1, Ym-1 and TGF-β) mRNA expression was notably elevated (Fig. 4G and H). The *in vitro* tests showed that NPs could change macrophage phenotype from M1 into M2. In addition, we have assessed peritoneal macrophages intracellular ROS by flow cytometry after different doses of NPs treatment (20 μg/mL, 50 μg/mL, 100 μg/mL), the flow cytometry and qRT-PCR results confirmed that the conversion of M1 to M2 macrophages in a dose-dependent manner (Fig. S9). The *in vivo* verification of this transition was conducted using immunofluorescence assay of lung tissues. The F4/80 used to sign the entire macrophages in CLP mice. After PDA NPs and PDA-PEI NPs treatment, Arg-1 expression rose whereas iNOS expression fell when compared the CLP with NPs groups (Fig. S10), suggesting that PDA-PEI NPs treatment converted M1 macrophages into M2 macrophages.

### 3.6 Anti-sepsis therapeutic efficacy in CLP induced sepsis

**Fig. 5:**
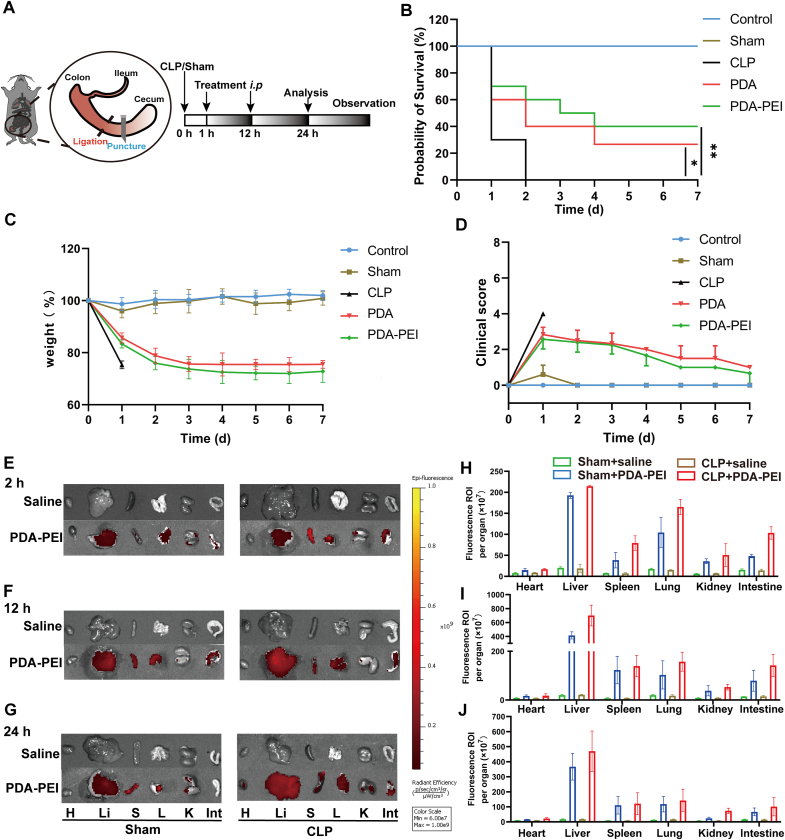
Therapeutic impact on septic mice. (A) Timetable for the model of CLP-induced sepsis experiments. (B) Survival rate. n = 10. (C) Body weight. n = 10. (D) Clinical score. n = 10. (E–G) Fluorescence pictures taken *ex vivo* of CLP and sham mice at 2 h (E), 12 h (F), and 24 h (G) following an intraperitoneal injection of DiR-labeled PDA-PEI NPs. (H–J) Quantification of the average fluorescence intensity of organs *ex vivo* of CLP and sham mice at 2 h (H), 12 h (I), and 24 h (J). n = 3. (∗*p* < 0.05, ∗∗*p* < 0.01).

Cecal ligation and puncture (CLP) model is one of the popular sepsis models, which leads to acute inflammation, multiple organ failure and cytokine storm [47]. In order to confirm the sepsis therapeutic effectiveness of NPs, we used C57/BL6 mice to create a CLP model. PDA NPs and PDA-PEI NPs at a dosage of 10 mg/kg were given intraperitoneally to mice at 1 h and 12 h following the build of CLP model (Fig. 5A). For seven days in a row, the survival percentage, body weight, and clinical ratings of mice were noted (Fig. 5B-D). All untreated mice succumbed less than 48 h. The mice treated with PDA NPs showed an improvement in survival to 20 %, and the PDA-PEI NPs group had the greatest survival rate of 40 % (Fig. 5B). The tissue retention duration of PDA-PEI NPs may be responsible for above outcomes. By simultaneously scavenging various inflammatory mediators in sepsis, these NPs may help restore immunological homeostasis and minimize multiorgan damage in mice. Surviving mice's body weight showed recovery after therapy (Fig. 5C) and the clinical ratings dramatically decreased (Fig. 5D), showing that the physical condition of mice improved.

The biodistribution of PDA-PEI NPs were assessed using fluorescent DiR-labeled PDA-PEI NPs and fluorescence imaging *ex vivo*. For fluorescence imaging, the NPs were administered 1 h after CLP, and the intestine and main organs were collected 2 h, 12 h, and 24 h later (Fig. 5E-G). Lung and intestinal fluorescence intensity of CLP mice in the NPs group peaked at 12 h and decreased thereafter. PDA-PEI NPs displayed extended preservation in the main organs and the inflamed intestine (Fig. 5H-J), promising for extended defense from sepsis. According to the results, the liver fluorescence intensity was the highest at all of three different time points (2 h, 12 h, 24 h) after CLP in the CLP + PDA-PEI group, and reach the peak at 12 h, then gradually decrease, so we reasonably speculated that the NPs were mainly cleared by the liver.

**Fig. 6:**
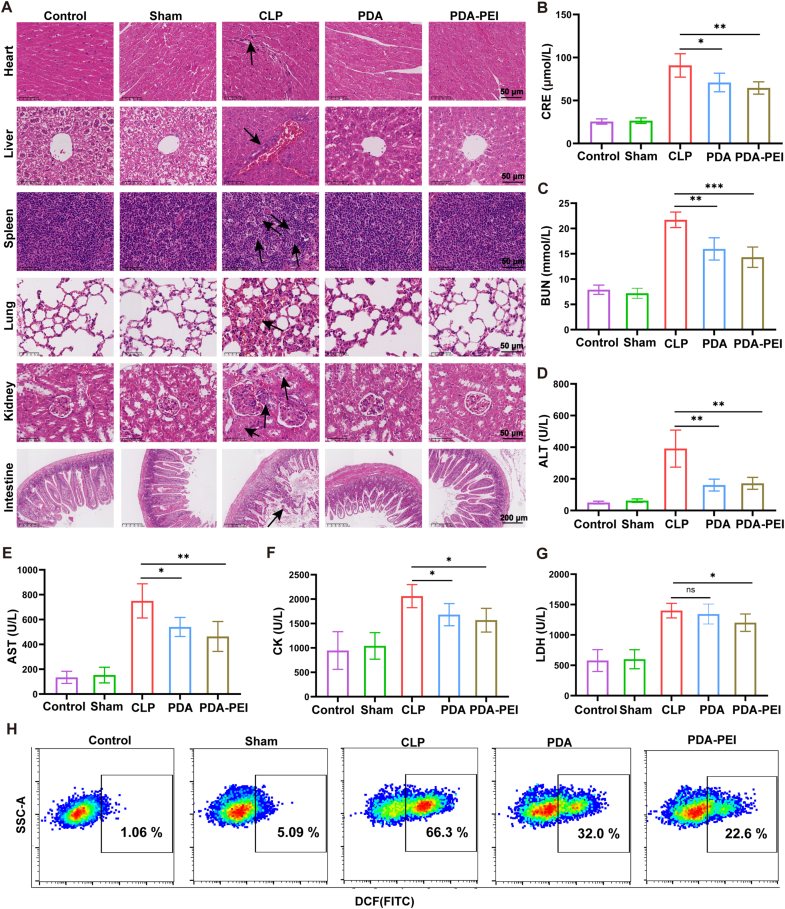
PDA-PEI NPs lessen failure of various organs in septic mice. (A) Tissues from the heart, liver, spleen, lung, kidney and intestine were stained with H&E and examined. Arrows are used to indicate various depictions of organ injury, including inflammatory cell infiltration in the heart and liver, thickened interalveolar septum in the lung, necrotic detachment of renal tubular epithelial cells and edema in the kidney and intestinal epithelial cell necrosis and shedding. Scale bar = 50 μm and 200 μm. (B–G) Blood serum biochemistry parameters CRE (B), BUN (C), ALT (D), AST (E), CK (F) and LDH (G) were measured 24 h after CLP. n = 5. (H) Assess the ROS levels of intraperitoneal cells by flow cytometry 24 h after CLP. (∗*p* < 0.05, ∗∗*p* < 0.01, ∗∗∗*p* < 0.001).

Given that harsh sepsis frequently results in multiple organs damage, we performed histological and biochemical investigations on mice handled with NPs 24 h after CLP [48]. H&E-stained sections of the heart, liver, spleen, lung, kidney and intestine in septic mice showed typical multiple organ damage. We observed inflammatory cell infiltration in the heart and liver, thickened interalveolar septum in the lung, necrotic detachment of renal tubular epithelial cells and edema in the kidney and intestinal epithelial cell necrosis and shedding (Fig. 6A). PDA-PEI NPs markedly reduced the elevated blood levels of CRE (Fig. 6B), BUN (Fig. 6C), ALT (Fig. 6D), AST (Fig. 6E), CK (Fig. 6F), and LDH (Fig. 6G), in accordance with these organ histopathological alterations. The results obtained *in vitro* and *in vivo* hold together.

**Fig. 7:**
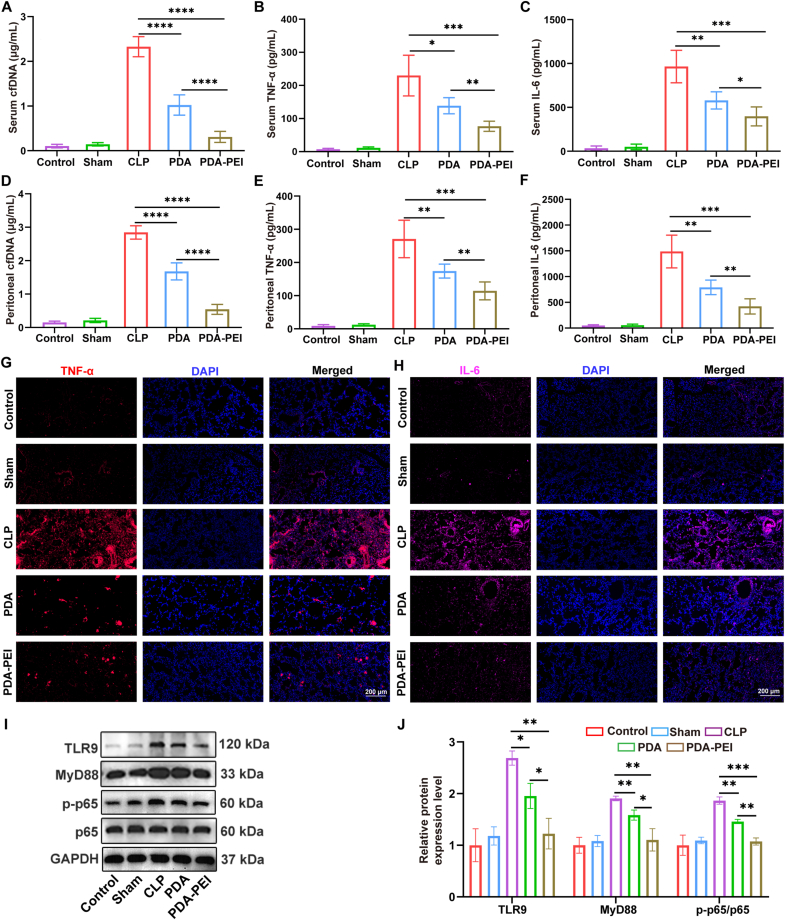
PDA-PEI NPs alleviate inflammation. (A) Serum cfDNA level 24 h after CLP. n = 5. (B) The level of serum inflammatory factors TNF-α. n = 5. (C) The level of serum inflammatory factors IL-6. n = 5. (D) Peritoneal cfDNA level 24 h after CLP. n = 5. (E) The level of peritoneal inflammatory factors TNF-α. n = 5. (F) The level of peritoneal inflammatory factors IL-6. n = 5. (G and H) Lung tissues immunofluorescence of inflammatory factors TNF-α (G) and IL-6 (H) 24 h after CLP. Scale bar = 200 μm. (I and J) Protein expression for TLR9, MyD88, p-p65 and p65 was applied to evaluate by western blot, the internal reference is GAPDH. n = 3. (∗*p* < 0.05, ∗∗*p* < 0.01, ∗∗∗*p* < 0.001, ∗∗∗∗*p* < 0.0001).

We then evaluated the ROS levels of each treatment group. Compared with the untreated CLP group, PDA NPs and PDA-PEI NPs treatment reduced the proportion of cells with higher ROS levels in the abdominal cavity (Fig. 6H and S11), indicating a relief of inflammation in this area. Compared with PDA NPs, PDA-PEI NPs treatment can significantly reduce ROS. We also investigated how the NPs affected cfDNA and inflammatory cytokines during sepsis. In contrast to the sham and control groups, the plasma cfDNA level of untreated CLP group was noticeably higher. The results show that treatment with PDA-PEI NPs decreased the amount of cfDNA in serum and peritoneal fluid (Fig. 7A and D), indicating excellent scavenging capability of cfDNA by the NPs *in vivo*. Surprisingly, PDA NPs decreased the amount of cfDNA in serum as well, perhaps as a result of the therapeutic effects reducing the discharge of cfDNA from injured cell. Since one of the main effects of TLR activation is the production of cytokines, we detected the level of TNF-α and IL-6, which are released mostly by activated immune cells. TNF-α and IL-6 levels in CLP group were higher than the control and sham group. Conversely, PDA NPs as well as PDA-PEI NPs therapies resulted in reduced the level of inflammatory cytokines in serum (Fig. 7B and C) and peritoneal fluid (Fig. 7E and F). PDA-PEI NPs exhibited a better diminution in cytokines compared to PDA NPs, presumably as a result of improved cfDNA scavenging ability. Sepsis patients frequently have acute respiratory distress syndrome or severe lung damage, which is the leading cause of mortality [49]. Immunofluorescence labeling of TNF-α and IL-6 in mice lung tissues further confirmed that CLP group exhibited elevated TNF-α and IL-6 expression (the red and pink areas represent positive expression) (Fig. 7G and H).

Then we elucidate how cfDNA contributes to the development of sepsis and investigate whether cfDNA-mediated TLR9 pathway activation results in the release of proinflammatory cytokines during sepsis. We harvested peritoneal macrophages from septic mice and extracted their proteins. Because of the TLR9-MyD88-NF-κB pathway is a common routing relate to cfDNA-induced TLR9 activation, we explored whether eliminating of cfDNA affects the expression of elements of this routing (Fig. 7I and J). The peritoneal macrophages of septic mice exhibited a large up-regulation of TLR9 and MyD88 expression, accompanied by a notable rise in p-p65/p65 levels, which was significantly suppressed by PDA-PEI NPs therapy. These results validated our envisagement and indicated that PDA-PEI NPs alleviate the inflammatory cytokine storm through the TLR9-MyD88-NF-κB routing cascade as sepsis progresses.

### 3.7 PDA-PEI NPs shows excellent biosafety *in vivo*

NPs have important clinical translational uses that require careful evaluation of biosafety. In this instance, PDA NPs, PEI and PDA-PEI NPs were given to healthy mice at a dosage (20 mg/kg) double the therapeutic level. Then, blood was drawn from their eyes for blood biochemical and blood cell count analysis after 24 h. Following euthanasia, major organs including the heart, liver, spleen, lung, kidney and intestine of mice were removed for histomorphologic pathological examination. Abnormal blood biochemical index, abnormal blood cell count and tissue injury were observed in PEI group. PDA NPs, and PDA-PEI NPs groups did not significantly alter the levels of any serum biochemical markers or blood cell counts (Figs. S12 and S13). To summarize, the biosafety of NPs *in vivo* was found to be favorable, indicating that they might be safely used in clinical settings.

## 4 Discussion

Sepsis is an increasingly severe systemic inflammatory response syndrome (SIRS) characterized by an amount of secreted inflammatory cytokines and subsequent organ failure, with a high mortality rate [50]. Due to the involvement of multiple inflammatory cytokines and cells in the inflammatory microenvironment of sepsis, treatment strategies targeting only a single pathogenic factor have limited efficacy [51]. The complex inflammatory microenvironment of sepsis poses significant challenges to the development of effective therapies. Recent evidence suggests that inflammatory dysregulation is closely related to the development of sepsis, whose causes include hyperactivation of TLR, massive accumulation of ROS, and increased pro-inflammatory macrophages [[52], [53], [54]]. Thus, multifunctional therapies that simultaneously target these therapeutic factors may be a productive direction for sepsis treatment. Clinically, patients with sepsis usually have pathogens and elevated cfDNA released from infected host cells [55]. Our study also found increased circulating cfDNA levels in septic mice. As cfDNA activates inflammation by activating TLR9, clearing cfDNA or blocking the aberrant cfDNA-sensing pathway appears to be fruitful in ameliorating systemic inflammation in severe sepsis. In addition to cfDNA, ROS plays an important role in the inflammatory response in sepsis, large accumulation of ROS cause damage to tissues and organs through oxidative stress, and ROS also acts as a second messenger in multiple inflammatory pathways [56]. Chen et al. [57] used multifunctional tea polyphenols nanoparticles to scavenge multiple reactive oxygen and nitrogen species (RONS) and excessive pyroptosis, thus effectively alleviating inflammation. In addition, we also observed that immunofluorescence analysis of the lung tissue of mice with sepsis, there was a significant increase in M1 macrophage markers, M1 macrophages are a type of pro-inflammatory immune cell that secretes numerous inflammatory cytokines, which contribute to the progression of sepsis. Inhibiting the expansion of M1 macrophages in the inflammatory microenvironment or transitioning them into M2 macrophages is an effective anti-inflammatory approach.

In order to demonstrate the favorable therapeutic effect of the multifunctional nanoparticle in sepsis. Here, we constructed a multifunctional nanoparticle PDA-PEI NPs based on PDA and PEI. On the one hand, leveraging the antioxidant and immune regulatory capacities of PDA, it diminishes pro-inflammatory M1 macrophages while scavenging ROS. On the other hand, by employing PEI, which carries a plethora of positive charges, the nanoparticle can capture negatively charged cfDNA and prevent its activation of TLR9, thereby inhibiting inflammatory response. Our *in vivo* and *in vitro* results also demonstrated the therapeutic effect of this multifunctional nanoparticle for sepsis. PDA-PEI NPs reduced the expression of cfDNA and TLR9-MyD88-NF-κB signaling pathway in serum and peritoneal fluid of septic mice, which leads to the decrease of inflammatory cytokines in septic mice. Moreover, immunofluorescence analysis of lung tissue demonstrated that PDA-PEI nanoparticles effectively downregulated the expression of iNOS, a marker for M1 macrophages, while upregulating the expression of Arg-1, a marker for M2 macrophages. These findings suggest that PDA-PEI NPs promote macrophage polarization and can shift M1 macrophages towards an anti-inflammatory M2 phenotype. The immunofluorescence of lung tissue inflammatory cytokines TNF-α and IL-6 indicates the anti-inflammatory ability of PDA-PEI NPs. The ROS scavenging experiments further validated the robust ROS scavenging effect of PDA-PEI NPs. It is anticipated that the utilization of PDA-PEI NPs, which exhibits enhanced removal of inflammatory mediators, will lead to improved therapeutic outcomes compared to using PDA NPs.

In summary, we have constructed a multifunctional nanoparticle that inhibits cfDNA stimulation of TLR9-mediated proinflammatory responses, reduces massive ROS accumulation in inflammatory cells and promotes polarization of M1 to M2 macrophages. In sepsis mice model, PDA-PEI NPs with good accumulation and retention behavior in inflamed tissues can effectively reduce septic death and ameliorate multiple organ damage. Cationic nanoparticles usually have biological toxicity, here we by adjusting the molecular weight and proportion of PEI to reduce the toxicity of nanoparticle to a suitable range. *In vivo* and *in vitro* experiments also demonstrated that PDA-PEI NPs had good safety, good accumulation and retention behavior in inflammatory tissues and organs, and could improve survival rate and reduce multi-organ damage. These results demonstrate that multifunctional nanoparticles are a promising therapeutic approach for sepsis with broad clinical applications in sepsis and fatal inflammatory diseases.

## 5 Conclusion

This study presents an innovative approach for the treatment of sepsis by developing cationic nanoparticles (NPs) with excellent DNA capture, anti-inflammatory and ROS scavenging properties. The cfDNA-induced TLR9 activation and NF-κB signaling were inhibited by the PDA-PEI NPs. PDA-PEI NPs have been shown to be beneficial in lowering multiple organ injury and raising the survival rate of mice suffering from sepsis *in vivo* tests. Furthermore, PDA-PEI NPs promote the conversion of M1 macrophages into M2 macrophages with anti-inflammatory effect. The inflammation control of PDA-PEI NPs increases the possibility of using them to treat severe conditions. Moreover, this strategy offers a possible way to treat inflammatory illnesses that have polarized M1 macrophages and increased cfDNA levels in common.

## CRediT authorship contribution statement

**Wenjie Xi:** Writing – original draft, Validation, Methodology, Investigation, Formal analysis, Data curation. **Weijie Wu:** Validation, Investigation, Funding acquisition, Formal analysis, Data curation. **Lili Zhou:** Validation, Investigation. **Qi Zhang:** Validation, Methodology, Data curation. **Shushu Yang:** Investigation, Formal analysis. **Lihong Huang:** Investigation, Formal analysis. **Yijun Lu:** Validation, Investigation. **Jing Wang:** Validation, Investigation. **Xinjin Chi:** Funding acquisition, Formal analysis, Data curation. **Yang Kang:** Writing – review & editing, Supervision, Funding acquisition, Data curation.

## Ethics approval and consent to participate

All animal studies were performed with the approval of the ethical review board at the Committee of Sun Yat-sen University has authorized all animal studies (SYSU-IACUC-2021-000559).

## Declaration of competing interest

The authors declare that they have no known competing financial interests or personal relationships that could have appeared to influence the work reported in this paper.

## Data Availability

Data will be made available on request.
