# Supplementary material for: Multifunctional nanoparticles confers both multiple inflammatory mediators scavenging and macrophage polarization for sepsis therapy
Source: Mater Today Bio. 2024 Dec 20;30:101421. doi: 10.1016/j.mtbio.2024.101421 (PMC11732566; doi:10.1016/j.mtbio.2024.101421)
Supplement: Multimedia component 1 [file mmc1.docx]

**Multifunctional polydopamine nanoparticles confers both multiple inflammatory mediators scavenging and macrophage polarization for sepsis therapy**

Wenjie Xi^a,b,1^, Weijie Wu^a,b,1^, Lili Zhou^a,1^, Qi Zhang^a^, Shushu Yang^b^, Lihong Huang^c^, Yijun Lu^a^, Jing Wang^a^, Xinjin Chi^a,∗∗^, Yang Kang^b,∗^

a. Surgical Anesthesia Center, the Seventh Affiliated Hospital of Sun Yat-sen University, Shenzhen, 518107, China

b. Scientific Research Center, the Seventh Affiliated Hospital of Sun Yat-sen University, Shenzhen, 518107, China

c. Department of Orthopaedics, the Eighth Affiliated Hospital of Sun Yat-sen University, Shenzhen, 518033, China

^∗^Corresponding author. Scientific Research Center, the Seventh Affiliated Hospital of Sun Yat-sen University, Shenzhen, China.

^∗∗^Corresponding author. Surgical Anesthesia Center, the Seventh Affiliated Hospital of Sun Yat-sen University, Shenzhen, China.

^1^These authors equally contributed to the work.


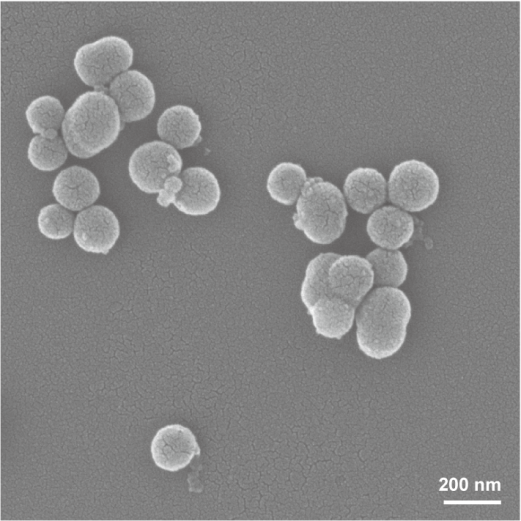


**Fig. S1.** SEM of PDA-PEI NPs. Scale bar = 200 μm.


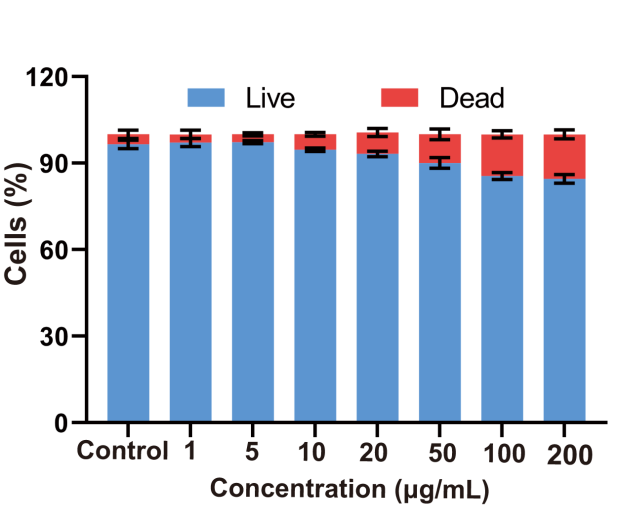


**Fig. S2.** Corresponding quantitative analysis of Fig. 2A. n = 3.


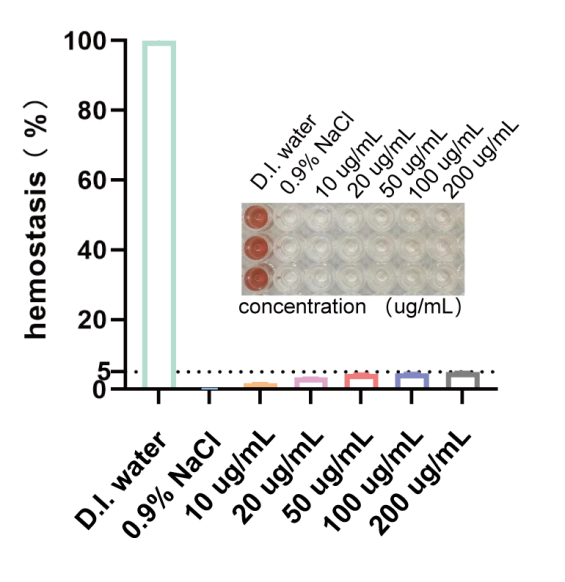


**Fig. S3.** Rate of hemolysis at various PDA-PEI NPs concentrations. n = 3.

**
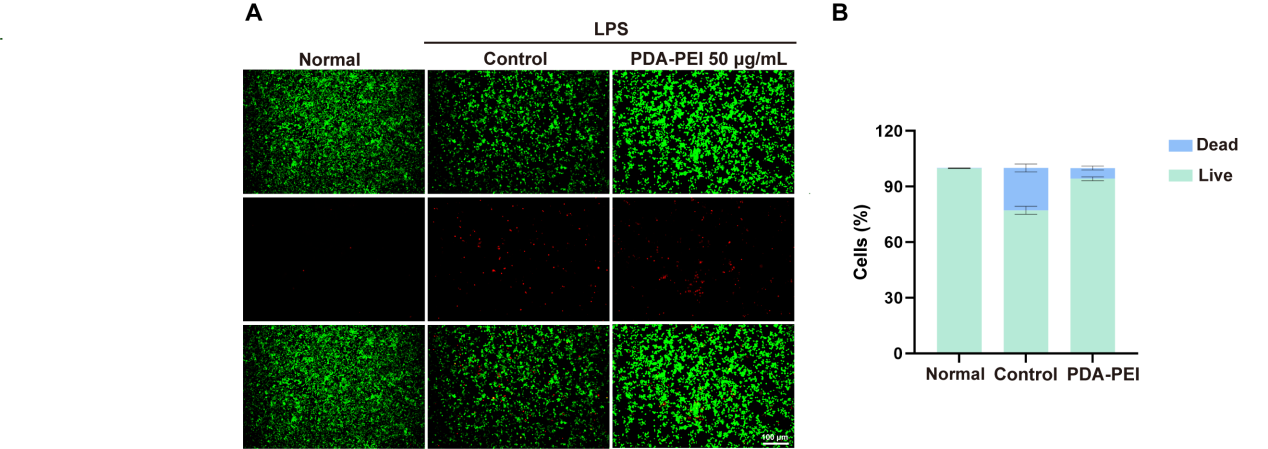
**

**Fig. S4.** (A) Fluorescence microscopy was applied to observe the live/dead labeling of LPS-treated macrophages (1 μg/mL, 4 h) and their 24 h incubation with PDA-PEI NPs (50 μg/mL). Scale bar = 100 μm. (B) Quantified outcomes of live/dead staining of LPS handled macrophages. n = 3.


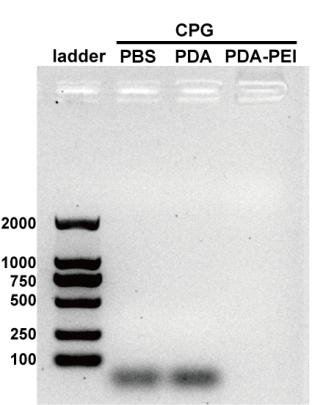


**Fig. S5.** DNA binding capacity of PDA NPs and PDA-PEI NPs by agarose gel electrophoresis.


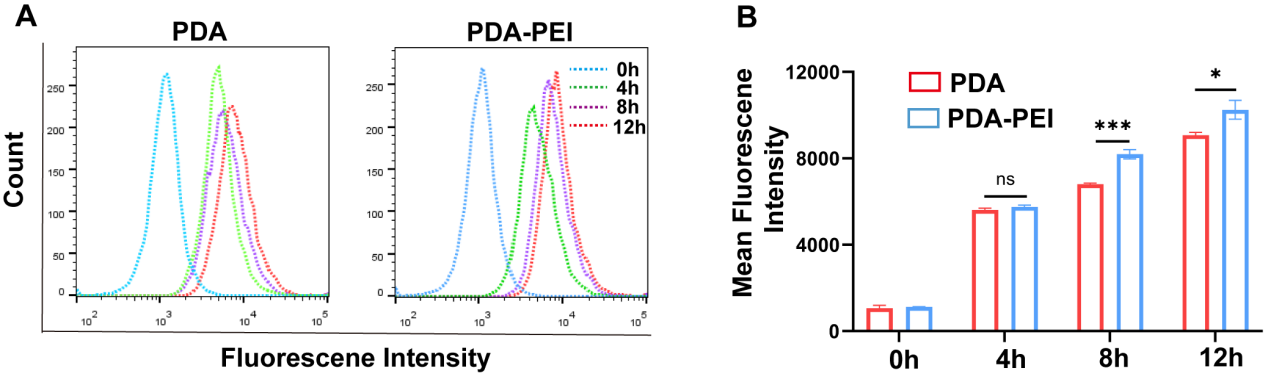


**Fig. S6.** (A) Cellular uptake of FITC-labeled PDA NPs and FITC-labeled PDA-PEI NPs in RAW264.7 cells at 4 h, 8 h, and 12 h using flow cytometry. (B) Corresponding quantitative analysis of Fig. S6A. n = 3. (**p* < 0.05, ****p* < 0.001)


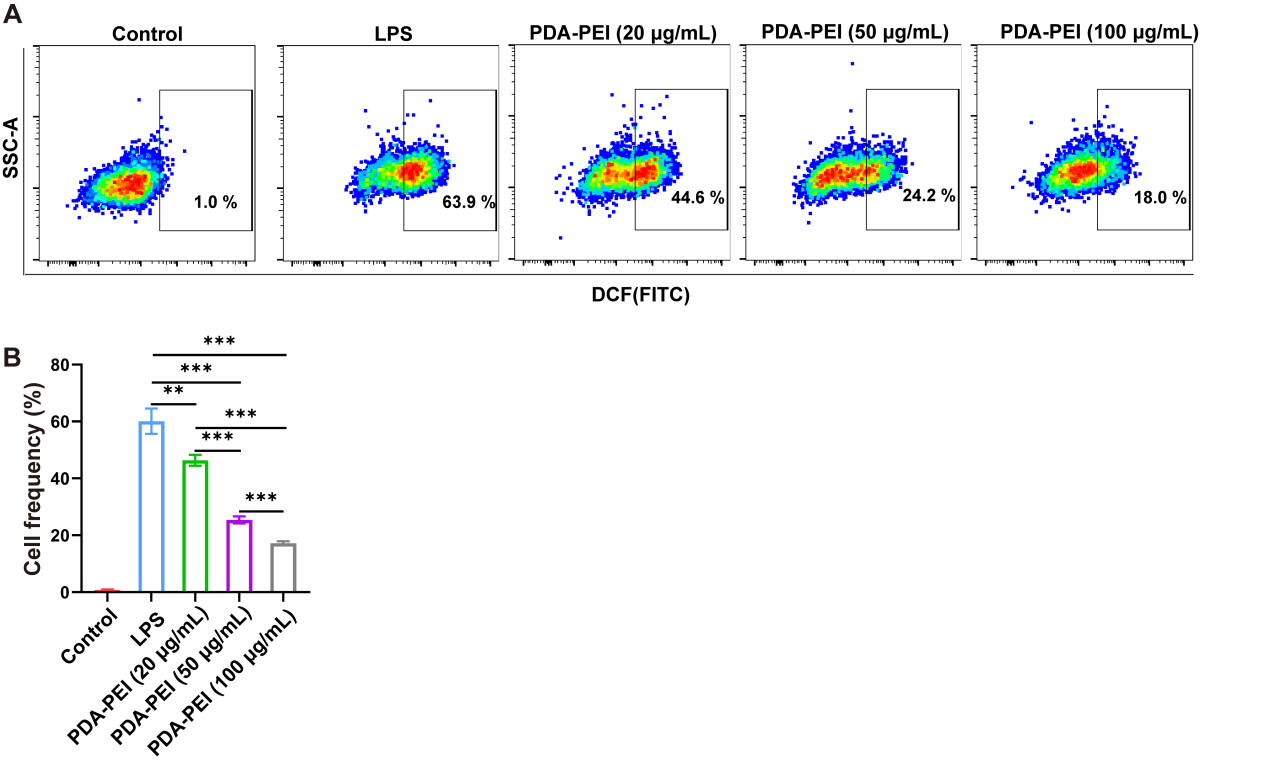


**Fig. S7.** Assessment of RAW264.7 cells intracellular ROS by flow cytometry after treatment of different concentrations PDA-PEI NPs.


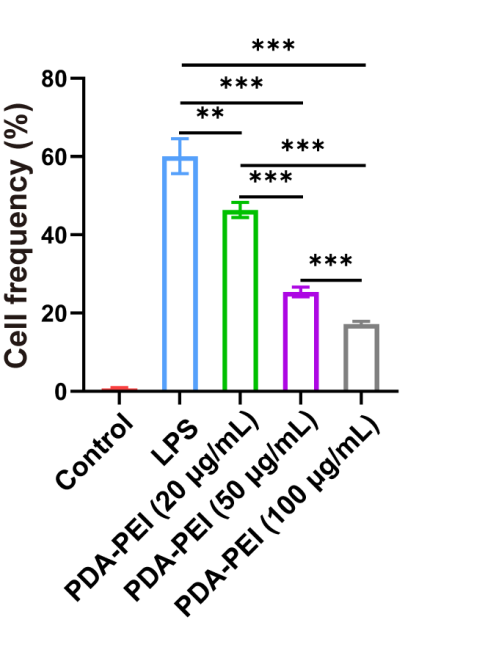


**Fig. S8.** Corresponding quantitative analysis of Fig. S7. n = 3. (***p* < 0.01, ****p* < 0.001)


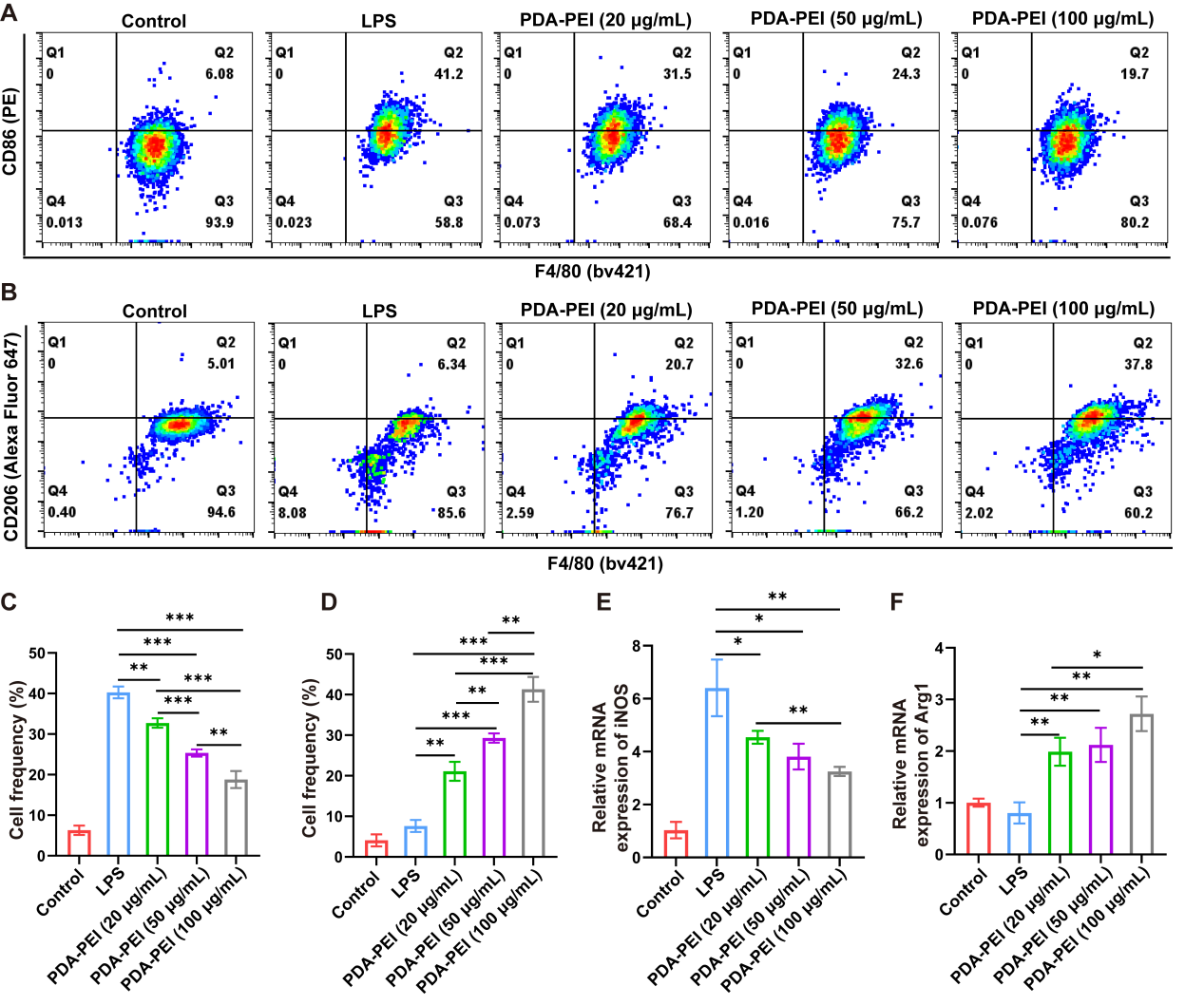


**Fig. S9.** NPs switches the M1-type macrophages to the M2-type macrophages in a dose-dependent manner. (A) Flow cytometry results showed that NPs of different concentrations could reduce M1-type macrophages. (B) Flow cytometry results showed that NPs of different concentrations could increase M2-type macrophages. (C) Corresponding quantitative analysis of Fig. S9A. n = 3. (D) Corresponding quantitative analysis of Fig. S9B. n = 3. (E and F) The mRNA expressions of M1 indicator (iNOS) and M2 indicator (Arg-1) were measured after treatment of NPs with different concentrations. n = 3. (**p* < 0.05, ***p* < 0.01, ****p* < 0.001)


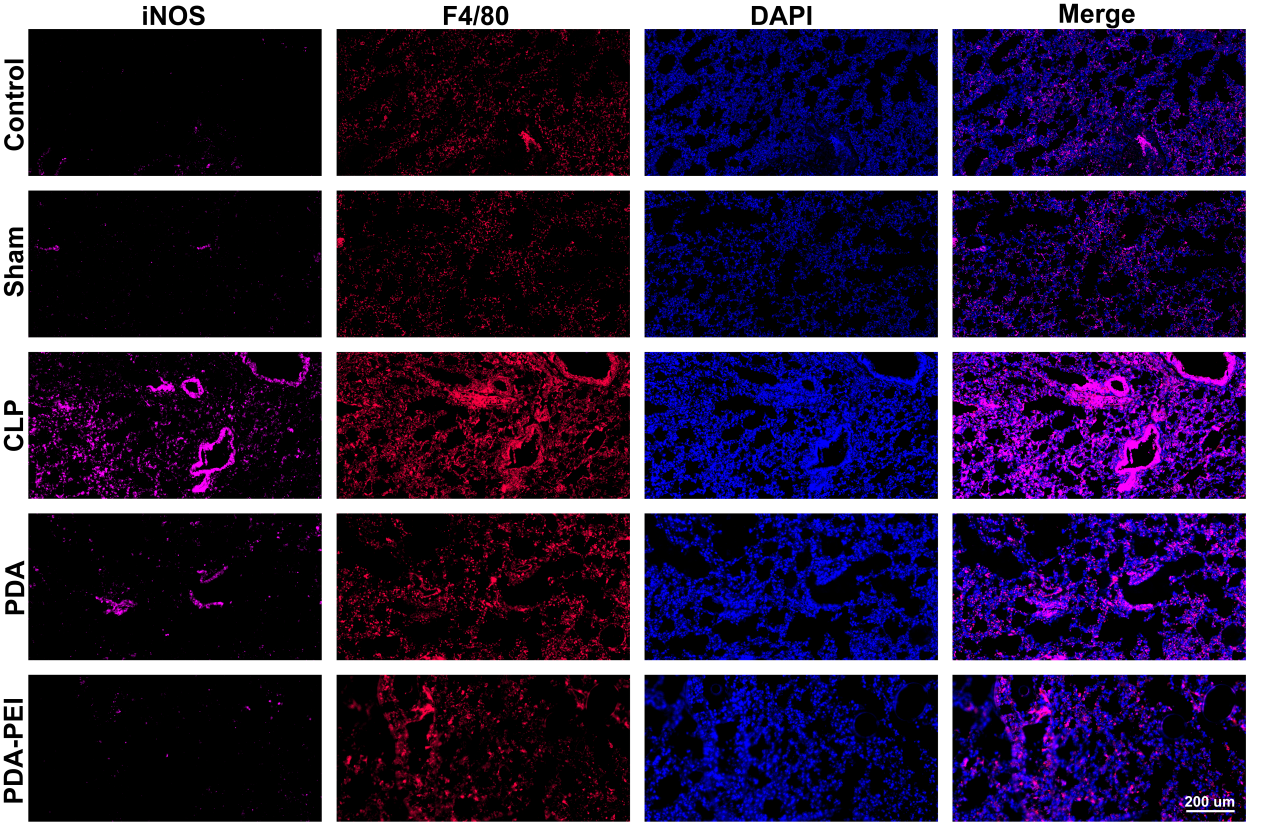

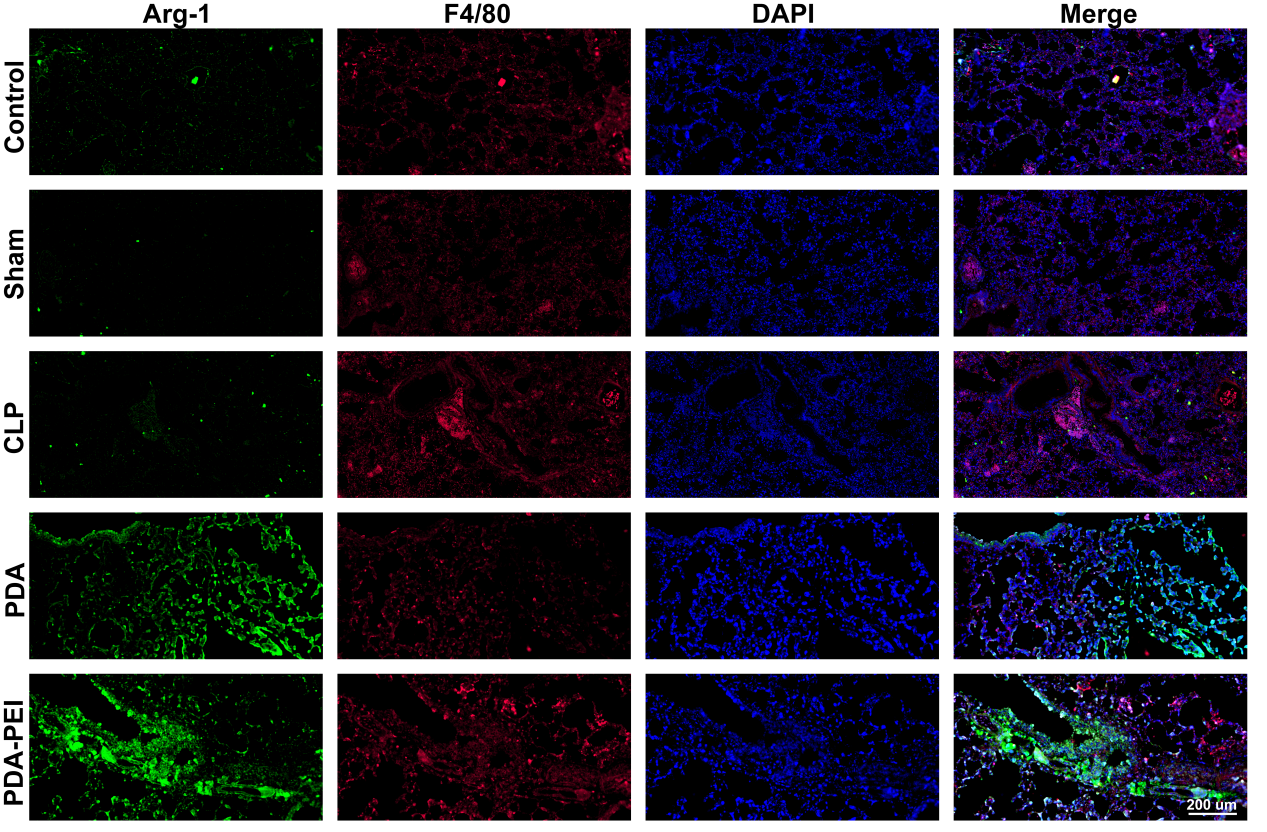


**Fig. S10.** Following a variety of treatments, the lung tissues were extracted from CLP mice, and lung slices were immunofluorescence stained with F4/80 (lung macrophages, red), iNOS (M1 marker, pink), Arg-1 (M2 marker, green) and DAPI (nuclei, blue). Representative images of random regions on each section were imaged using CLSM. Scale bar = 200 μm.

**
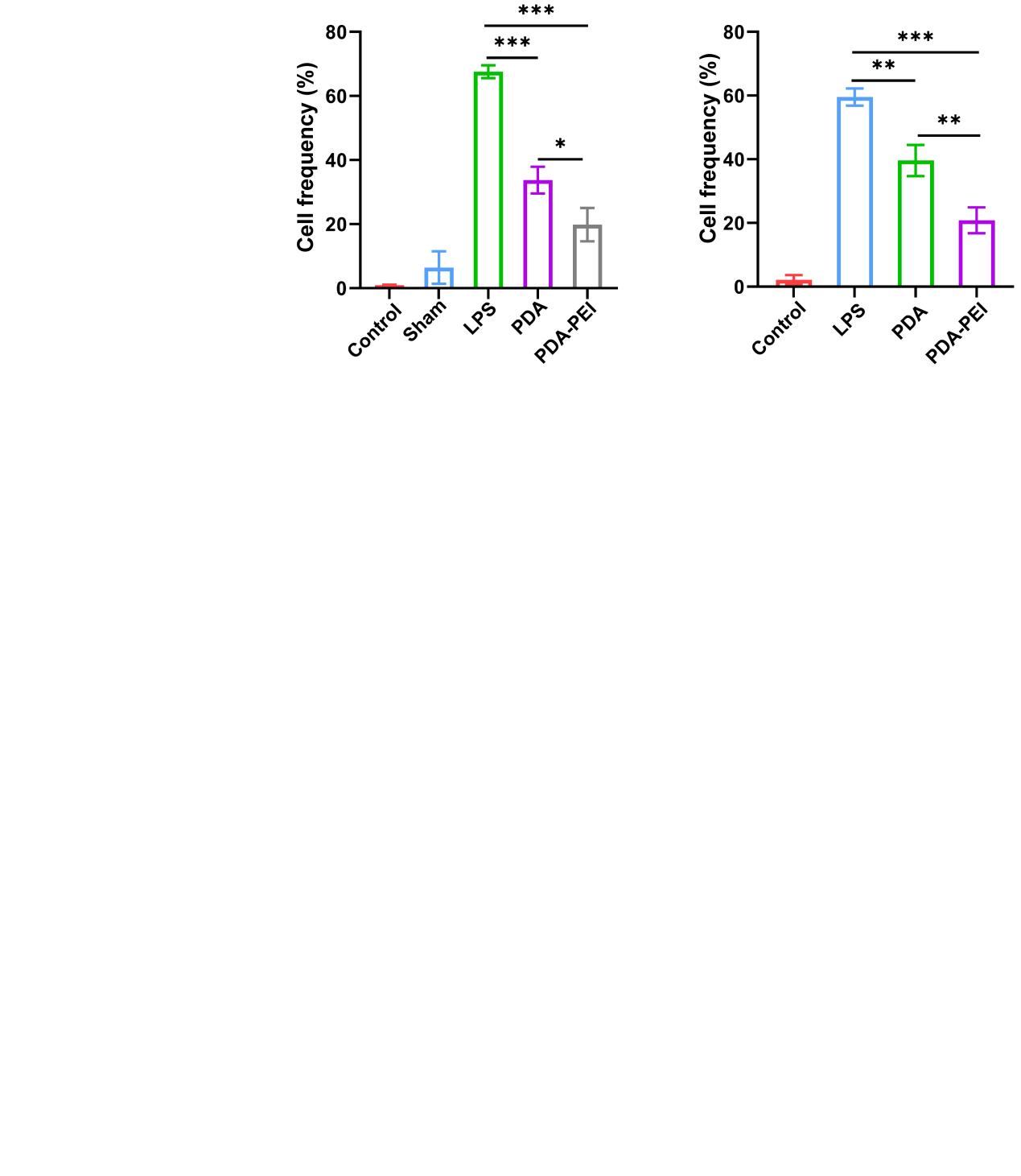
**

**Fig. S11.** Corresponding quantitative analysis of Fig. 6H. n = 3.

**
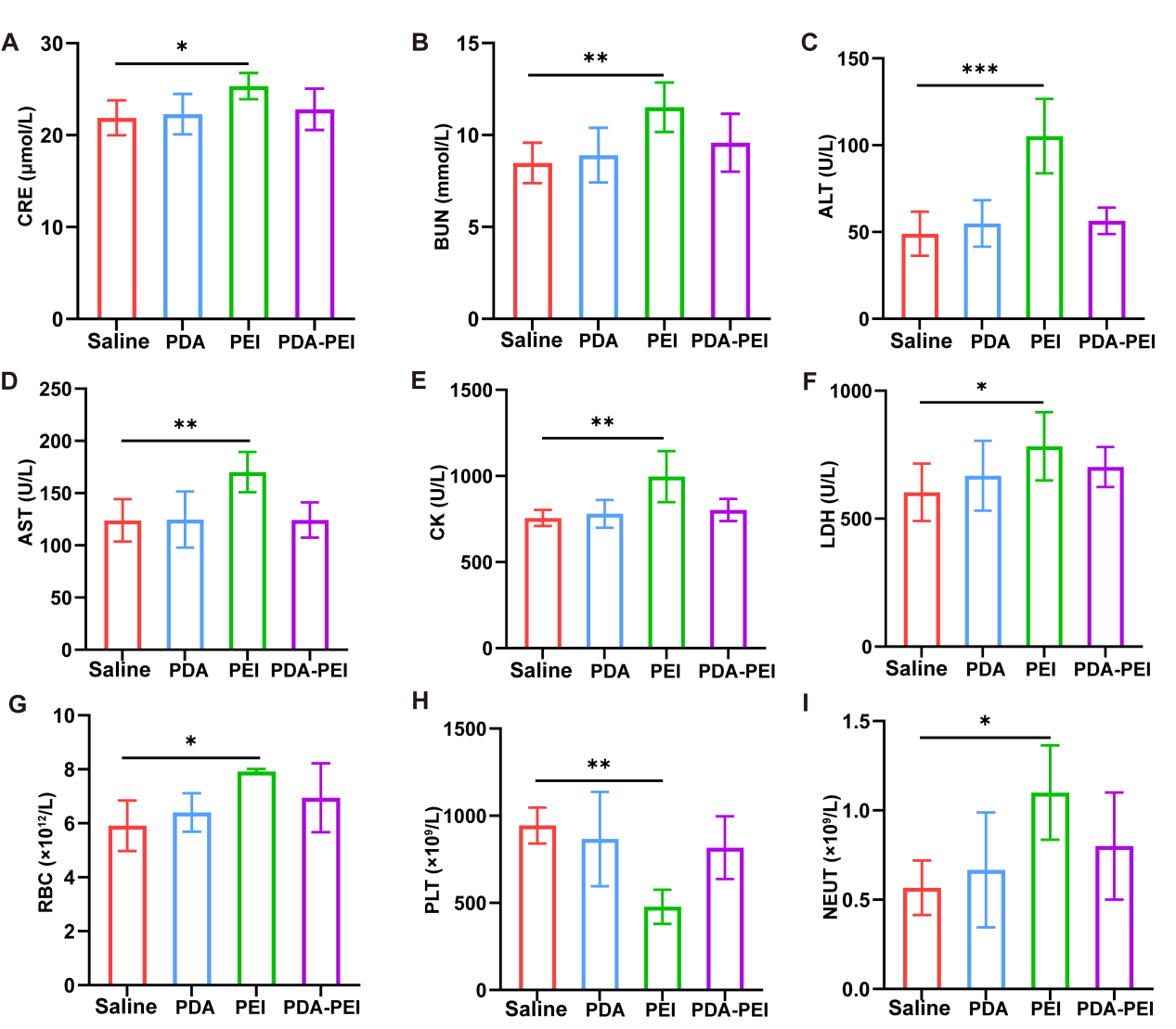
**

**Fig. S12.** PDA-PEI NPs shows lower toxicity than PEI *in vivo*. The mice were given intraperitoneal injections of NPs at a dosage (20 mg/kg) twice as therapeutic. (A) CRE, (B) BUN, (C) ALT, (D) AST, (E) CK, and (F) LDH were measured in serum after 24 h of exposure. (G) red blood cell (RBC) count, (H) platelet (PLT)) count and (I) neutrophil (NEUT) count in the blood were assessed after 24 h of exposure. n = 5. (**p* < 0.05, ***p* < 0.01, ****p* < 0.001)


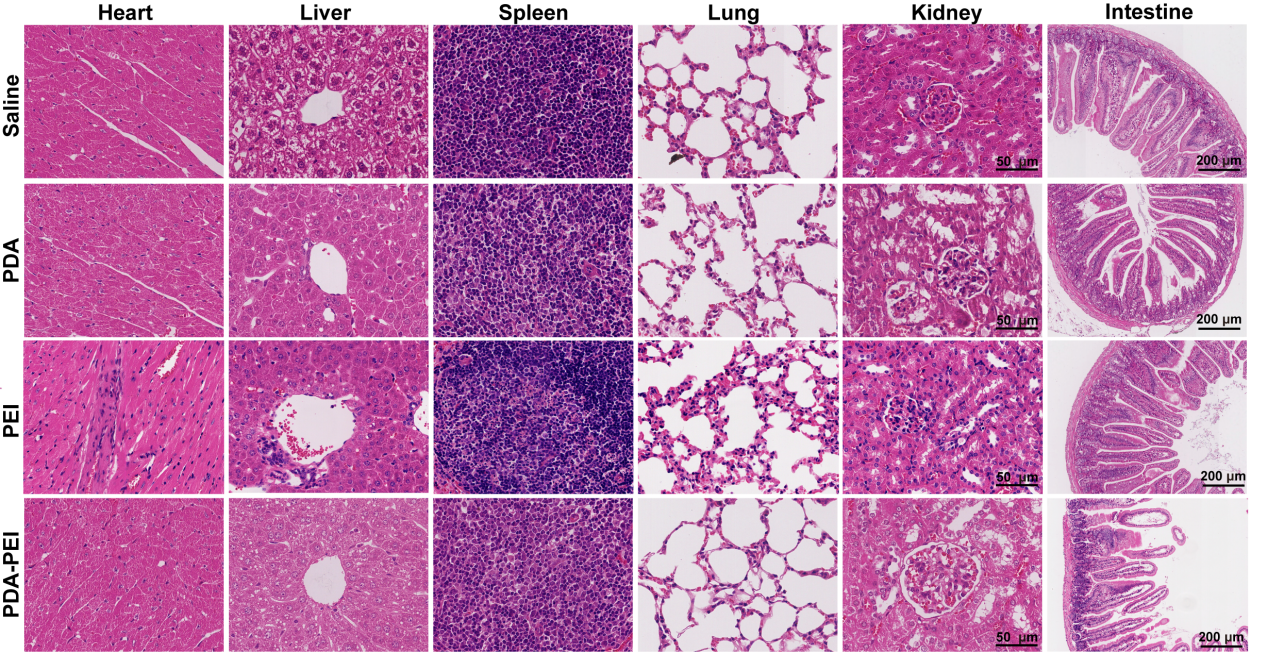


**Fig. S13.** H&E images obtained from the major organs of the PDA NPs, PEI, PDA-PEI NPs and saline-treated mice. Scale bar = 50 μm and 200 μm.

**Table S1.** Related information about antibodies.

| Catalog  Number | Product  Name | Species  Reactivity | Source |
| --- | --- | --- | --- |
| A19653 | NF-κB p65 | Rabbit | Abclonal, China |
| S536 | Phospho NF-κB p65 | Rabbit | Abclonal, China |
| A21905 | MyD88 | Rabbit | Abclonal, China |
| ab134368 | TLR9 | Mouse | Abcam, England |
| ab32527 | CCR7 | Rabbit | Abcam, England |
| ab19229 | Ym-1 | Rabbit | Abcam, England |
| ab183218 | TNF-α | Rabbit | Abcam, England |
| 5174 | GAPDH | Rabbit | Cell Signaling Technology, USA |
| 93668 | Arg-1 | Rabbit | Cell Signaling Technology, USA |
| 13120 | iNOS | Rabbit | Cell Signaling Technology, USA |
| GB11117 | IL-6 | Rabbit | Servicebio, China |
| GB113373 | F4/80 | Rabbit | Servicebio, China |
| SJ20-00 | CD86 | Rabbit | HUABIO, China |
| A11192 | CD206 | Rabbit | Abclonal, China |
| 553692 | PE Rat Anti-Mouse CD86 | Rabbit | BD, USA |
| 568808 | Alexa Fluor™ 647 Rat Anti-Mouse CD206 | Rabbit | BD, USA |
| 565411 | BV421 Rat Anti-Mouse F4/80 | Rabbit | BD, USA |
| 156603 | TruStain FcX™ (anti-mouse CD16/32) | Rabbit | BD, USA |
|  |  |  |  |

**Table S2.** Primers sequences for mRNAs

| Gene | Sequence (5’ to 3’, forward) | Sequence (3’ to 5’, reverse) |
| --- | --- | --- |
| iNOS | AGCCAAGCCCTCACCTACTT | TCTGCCTATCCGTCTCGTCC |
| TNF-α | AAGGCCGGGGTGTCCTGGAG | AGGCCAGGTGGGGACAGCTC |
| Arg-1 | ACAAGACAGGGCTCCTTTCAG | GGCTTATGGTTACCCTCCCG |
| TGF-β | GCTGCGCTTGCAGAGATTA | AGCCCTGTATTCCGTCTCCT |
| CCR7 | GGTGGCTCTCCTTGTCATTTTC | AGGTTGAGCAGGTAGGTATCCG |
| IL-1β | GCTCATTGTGGGATTTCCAGCA | CCTCAGTGGCTCCTTCATTCAGAA |
| Ym-1 | GCAACTGTTCCTGAACTCAACT | ATCTTTTGGGGTCCGTCAACT |
| GADPH | CCCTTAAGAGGGATGCTGCC | TACGGCCAAATCCGTTCACA |
